# Growth factors and mechano-regulated reciprocal crosstalk with extracellular matrix tune the keratocyte–fibroblast/myofibroblast transition

**DOI:** 10.1038/s41598-023-37776-9

**Published:** 2023-07-13

**Authors:** Simon A. Pot, Zhe Lin, Jauye Shiu, Mario C. Benn, Viola Vogel

**Affiliations:** 1 Laboratory of Applied Mechanobiology, Department of Health Sciences and Technology, ETH Zurich, Vladimir-Prelog-Weg 4, 8093 Zurich, Switzerland; 2 Ruisi (Fujian) Biomedical Engineering Research Center Co Ltd, 26-1 Wulongjiang Road, Fuzhou, 350100 People’s Republic of China; 3 Graduate Institute of Biomedical Sciences, China Medical University, No. 91, Xueshi Rd, North District, Taichung City, Taiwan; 4 Ophthalmology Section, Vetsuisse Faculty, University of Zurich, Winterthurerstrasse 260, 8057 Zurich, Switzerland

**Keywords:** Mechanisms of disease, Molecular conformation, Regenerative medicine

## Abstract

Improper healing of the cornea after injury, infections or surgery can lead to corneal scar formation, which is associated with the transition of resident corneal keratocytes into activated fibroblasts and myofibroblasts (K–F/M). Myofibroblasts can create an extracellular matrix (ECM) niche in which fibrosis is promoted and perpetuated, resulting in progressive tissue opacification and vision loss. As a reversion back to quiescent keratocytes is essential to restore corneal transparency after injury, we characterized how growth factors with demonstrated profibrotic effects (PDGF, FGF, FBS, TGFβ1) induce the K–F/M transition, and whether their withdrawal can revert it. Indeed, the upregulated expression of αSMA and the associated changes in cytoskeletal architecture correlated with increases in cell contractility, fibronectin (Fn) and collagen matrix density and Fn fiber strain, as revealed by 2D cell culture, nanopillar cellular force mapping and a FRET-labeled Fn tension probe. Substrate mechanosensing drove a more complete K–F/M transition reversal following growth factor withdrawal on nanopillar arrays than on planar glass substrates. Using decellularized ECM scaffolds, we demonstrated that the K–F/M transition was inhibited in keratocytes reseeded onto myofibroblast-assembled, and/or collagen-1-rich ECM. This supports the presence of a myofibroblast-derived ECM niche that contains cues favoring tissue homeostasis rather than fibrosis.

## Introduction

Proper corneal wound healing requires a sequence of well-tuned biochemical and biophysical events to properly regenerate the damaged tissue^1,2^. The transparency of the cornea is tightly linked to its extracellular matrix (ECM) architecture which is assembled and maintained by resident keratocytes, i. e. specialized mesenchymal cells that minimize light scattering, form a network and have a characteristic dendritic shape. Native keratocytes in the unwounded cornea display low contractility and do not express filamentous actin (F-actin) stress fibers, nor do they assemble a notable Fn matrix^3^. The transition of resident keratocytes into activated wound fibroblasts and myofibroblasts is induced by growth factors released at wound sites (most notably Transforming Growth Factor β1 (TGFβ1) and Platelet-Derived Growth Factor (PDGF)), the extracellular matrix (ECM) composition of the wound bed (e.g. ED-A fibronectin (Fn)), and alterations of wound mechanics (e.g. tissue tension)^4^. The transition of resident keratocytes into activated wound fibroblasts or alpha smooth muscle actin (αSMA) expressing myofibroblasts in response to injury is referred to as keratocyte–fibroblast/myofibroblast (K–F/M) transition. K–F/M is marked by alterations in ECM production, cytoskeletal architecture, and αSMA expression to enhance cell traction forces needed to contract and finally close a wound^1,2,5,6^. After wound closure, a reversion back to the keratocyte phenotype is essential to restore corneal transparency.

A wounded epithelium is an important source of various K–F/M transition-inducing growth factors, including PDGF, fibroblast growth factor (FGF), and TGFβ1^6^. The concentrations of PDGF, FGF, and TGFβ1 in the wound environment decrease again once epithelial wound healing is completed^6^. This leads to a reduced K–F/M transition and increased myofibroblast apoptosis or phenotype reversal, facilitating regenerative stromal remodeling processes^6,7^. Myofibroblast disappearance from wound sites is necessary to prevent progressive scar formation. However, the ECM deposited by myofibroblasts during wound healing persists in scar tissue environments and affects resident cells long after the acute wound healing phase has subsided. The (mis)-instructive role of fibrotic ECM cues can therefore persist much longer than the influence of auto- and paracrine signals^8–10^. As such, even after the successful removal of scar tissue myofibroblasts, the presence of scar tissue ECM can create a niche in which fibrosis is promoted and perpetuated. This pathological ECM environment can push newly recruited cells towards the generation of new scar tissue^8–10^**.** Much like the fibrotic cancer associated ECM triggers the transition of normal tissue fibroblasts to the myofibroblast-like cancer-associated fibroblast phenotype^11^. Fibrosis has been studied extensively and is relatively well understood, especially regarding the mechanism of action of its biochemical components. However, significant knowledge gaps exist, specifically regarding the multitude of biophysical aspects that regulate the reciprocal ECM-cell interactions that drive tissue (re)modeling, thereby contributing either to fibrosis, or the disappearance of myofibroblasts from healing wound sites^12^.

Central to the healing of wounds is cell proliferation and the assembly of new ECM. Fibronectin is the first provisional ECM that is actively assembled by either platelets^13^, or activated fibroblasts and myofibroblasts, in response to corneal injury^14^. The amount and organization of Fn present in a wound is correlated to the stage of wound healing^1,15^. Fn provides both biochemical and mechanical cues for adherent cells at the early stages of wound healing^16^ and serves as template for the subsequent deposition of other ECM molecules, particularly of type I collagen^16–18^. As cells pull on Fn fibers, Fn domains can be stretched and partially unfolded by cell-generated forces, which can expose some binding sites that are buried in the native fold, while turning off others^18–21^. For example, Fn fiber stretching exposes cryptic Fn–Fn self-assembly sites^22^, which accelerates Fn fibrillogenesis, cross-linking and fiber bundling^19,22,23^. Whereas Fn fiber stretching decreases the binding affinity to collagen I, as the unstretched N-terminus of Fn provides a multivalent template for collagen-1 nucleation and fiber assembly^18^. Fn domain unfolding can also change the affinity of certain cell surface receptors^24,25^. The mechanobiology of the ECM can thus be dynamically regulated by alterations in cell contractility, allowing the ECM to function as a dynamic signaling reservoir that provides biochemical and biophysical guidance to the resident cells^19^. As such, ECM-induced cell responses are altered by wound healing myofibroblast-mediated changes in the global architecture and stiffness, and local composition and organization (e.g. Fn fiber stretching), of the ECM^9,10,19,26^.

However, little is known whether Fn fiber tension might correlate with or even regulate the K–F/M transition, or its reversal. Treatment with wound healing-associated growth factors like PDGF, FGF, and TGFβ1 initiates K–F/M phenotype transition and stimulates Fn fibrillogenesis, and matrix remodeling and contraction^5,27,28^. Because cellular forces can stretch Fn^19,21^, we asked here to what extent the treatment with and deprivation of wound healing-associated growth factors affects the ability of corneal keratocytes to generate forces, assemble a Fn matrix and stretch the Fn fibrils within it. Gaining insights into the mechanobiological functional regulation of ECM during wound healing is particularly important since Fn fiber stretching directly regulates further Fn fibrillogenesis and the subsequent initiation of collagen-1 assembly^18,19^, and myofibroblast differentiation^29^, thereby decisively influencing wound healing outcomes.

To ask whether the level of Fn fiber stretching and collagen fiber content within the surrounding matrix might influence myofibroblast phenotype switching, and to address the abovementioned questions, a unique combination of biochemical and physical assays was exploited to illuminate mechanoregulatory factors in corneal wound healing. Primary rabbit keratocytes were cultured in growth factor conditioned and deprived environments to tune keratocyte phenotypes in vitro that are representative of the initial and late phases of wound healing^27^. To ask how phenotypic changes relate to the ability to create traction forces, a nanopillar assay^30^ was used to probe cellular traction forces with subcellular resolution. To probe Fn fiber stretching in the ECM of conditioned keratocytes, fluorescence resonance energy transfer (FRET)-labeled Fn (Fn-FRET) was used as tension probe to evaluate the in situ molecular conformation of fibrillar Fn within the cell assembled ECM^31^. Finally, to study the influence of the ECM composition and Fn fiber tension on myofibroblast differentiation, keratocytes were reseeded in decellularized ECM scaffolds.

## Results

### Growth factor-conditioning induced a keratocyte to fibroblast/myofibroblast (K–F/M) transition with increased fibronectin and collagen-1 ECM assembly

Primary keratocytes from healthy rabbit eyes were cultured on glass cell culture substrates with adsorbed Fn in serum-free medium to maintain the native phenotype. Keratocytes were then exposed to 4-day cycles of stimulation with growth factors that are either protective of the keratocyte phenotype, e.g. IGF-1 (Insulin-like Growth Factor)^5,27,32^, or have demonstrated profibrotic/K–F/M transition driving effects, e.g. PDGF (Platelet-Derived Growth Factor), FGF (Fibroblast Growth Factor), FBS (fetal bovine serum), and TGFβ1 (Transforming Growth Factor β1). Cell and ECM morphologies were assessed by confocal fluorescence microscopy after (immuno)staining for F-actin, the myofibroblast marker αSMA, and Fn and collagen-1.

Control keratocytes assumed a stellate shape and growth factor conditioned keratocytes adopted either an elongated morphology with several long extensions (PDGF), or a fibroblast-like (FGF, FBS) or myofibroblast-like αSMA positive stress fiber-rich (TGFβ1) morphology (Supplementary Figs. S1 and S2), as previously described^28^. mRNA expression of selected keratocyte (keratocan, ALDH1a1) and myofibroblast (αSMA) markers confirmed the phenotype of the native keratocytes and of transformed (myo)fibroblast phenotypes, respectively (Supplementary Fig. S3 and Table S1).

**Figure 1:**
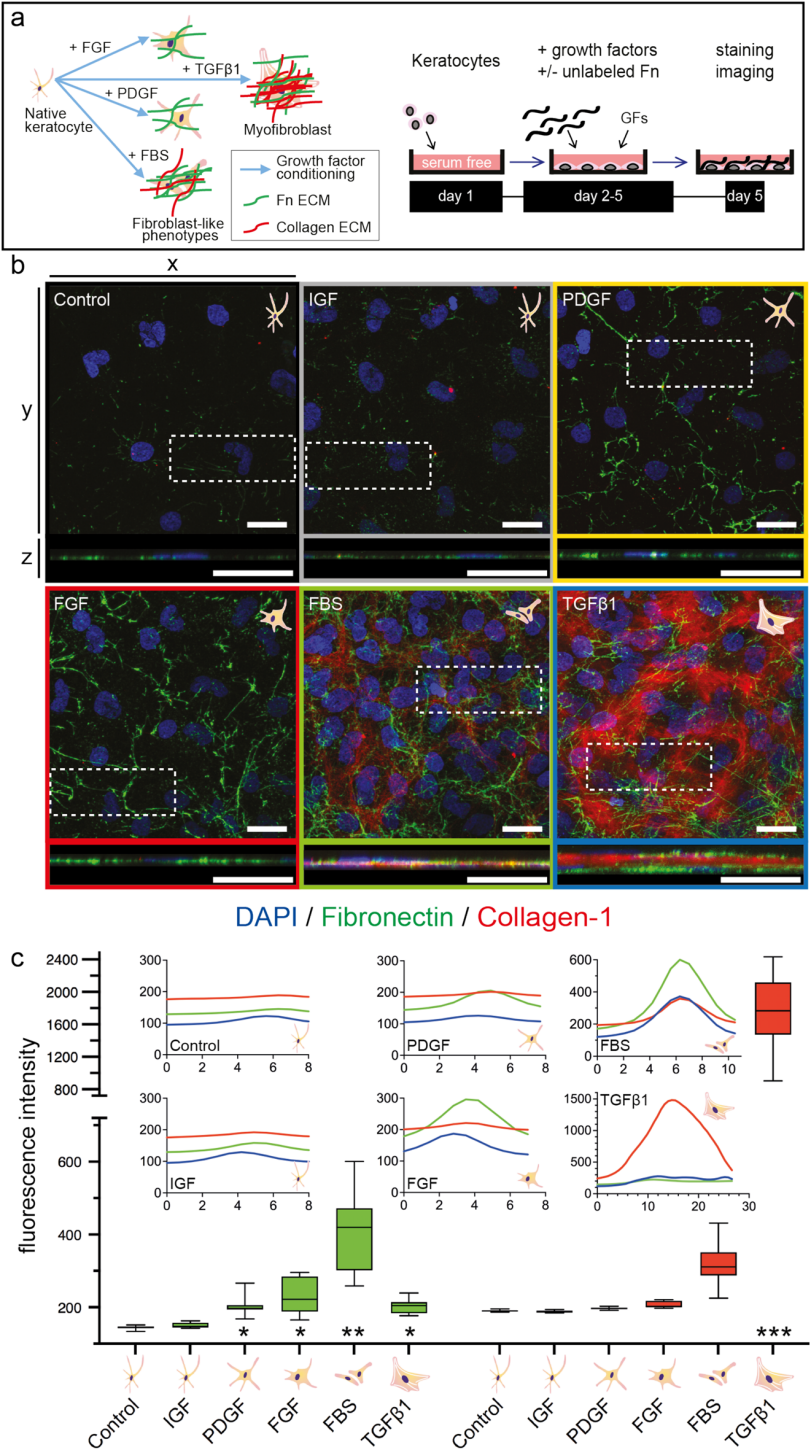
Fibronectin and Collagen-1 ECM assembly is increased for growth factor conditioned keratocytes. (**a**) brief overview of the keratocyte–fibroblast/myofibroblast (K–F/M) phenotype transition process in vitro and experimental timeline. One day of cell attachment, four days of growth factor exposure to various growth factors (IGF-1, PDGF, FGF, TGFβ1) or FBS in the presence of 50 mg/ml unlabeled Fn, fixation, Fn and collagen-1 staining and imaging. (**b**, **c**) effect of exposure to various growth factors on Fn and collagen-1 fibrillogenesis. Limited, fragmentary, pericellular Fn fibril assembly in native control and IGF-1-conditioned keratocytes. Significantly denser fibrillar Fn network assembly by PDGF, FGF and TGFβ1-conditioned keratocytes (*:*p* < 0.05–0.0001), with the densest fibrillar Fn ECM assembled by FBS-conditioned keratocytes (**:*p* < 0.05–0.0001). TGFβ1-conditioned keratocytes deposited significantly more collagen-1 (***:*p* < 0.0001), resulting in a 1.5–3 times thicker sample compared to any of the other phenotypes tested (**b**; **c**). The x–y views in (**b**) are maximum intensity z-projections of the entire 8–30 µm high image stacks, the x–z views are maximum intensity y-projections (100/512 slices) of the white boxed-in regions of interest. Green = fibronectin, red = collagen-1, blue = DAPI. Scale bars: 25 µm. The box plots in (**c**) demonstrate Fn (green) and Collagen-1 (red) fluorescence intensities for all cell phenotypes. Boxes signify medians, 25th and 75th percentiles. Whiskers represent the measurement range. The x-axes of the small graphs indicate sample thickness in µm. All y-axes indicate raw fluorescence intensities (gray values). FBS and TGFβ1 data were not plotted on the same scale as the data for the control, IGF-1, PDGF and FGF phenotypes. Statistical comparisons between phenotypes were performed via one-way ANOVA with Tukey’s multiple comparisons test (Collagen-1) and Kruskal–Wallis with Dunn’s multiple comparisons test (Fn), with significance set at *p* < 0.05 for all comparisons. The various phenotypes have been color coded in the relevant figures and graphs throughout the manuscript: native keratocyte control = black, IGF-1 = grey, PDGF = orange, FGF = red, FBS = green, TGFβ1 = blue.

PDGF, FGF, TGFβ1, and especially FBS conditioning increased metabolic activity (Supplementary Fig. S4), and activated Fn fibrillogenesis, resulting in a significantly denser Fn matrix surrounding the fibroblast and myofibroblast phenotypes compared to control and IGF-1 conditioned keratocytes (*p* < 0.05–0.0001) (Fig. 1). Collagen-1 fibril deposition was significantly increased following TGFβ1 conditioning (*p* < 0.0001) (Fig. 1). Our findings thus confirm and complement various previous reports^5,33,34^.

### FBS and TGFβ1 conditioned keratocytes display increased contractility with altered distribution of adhesive forces across the cell body, which is reversed after FBS and TGFβ1 deprivation

**Figure 2:**
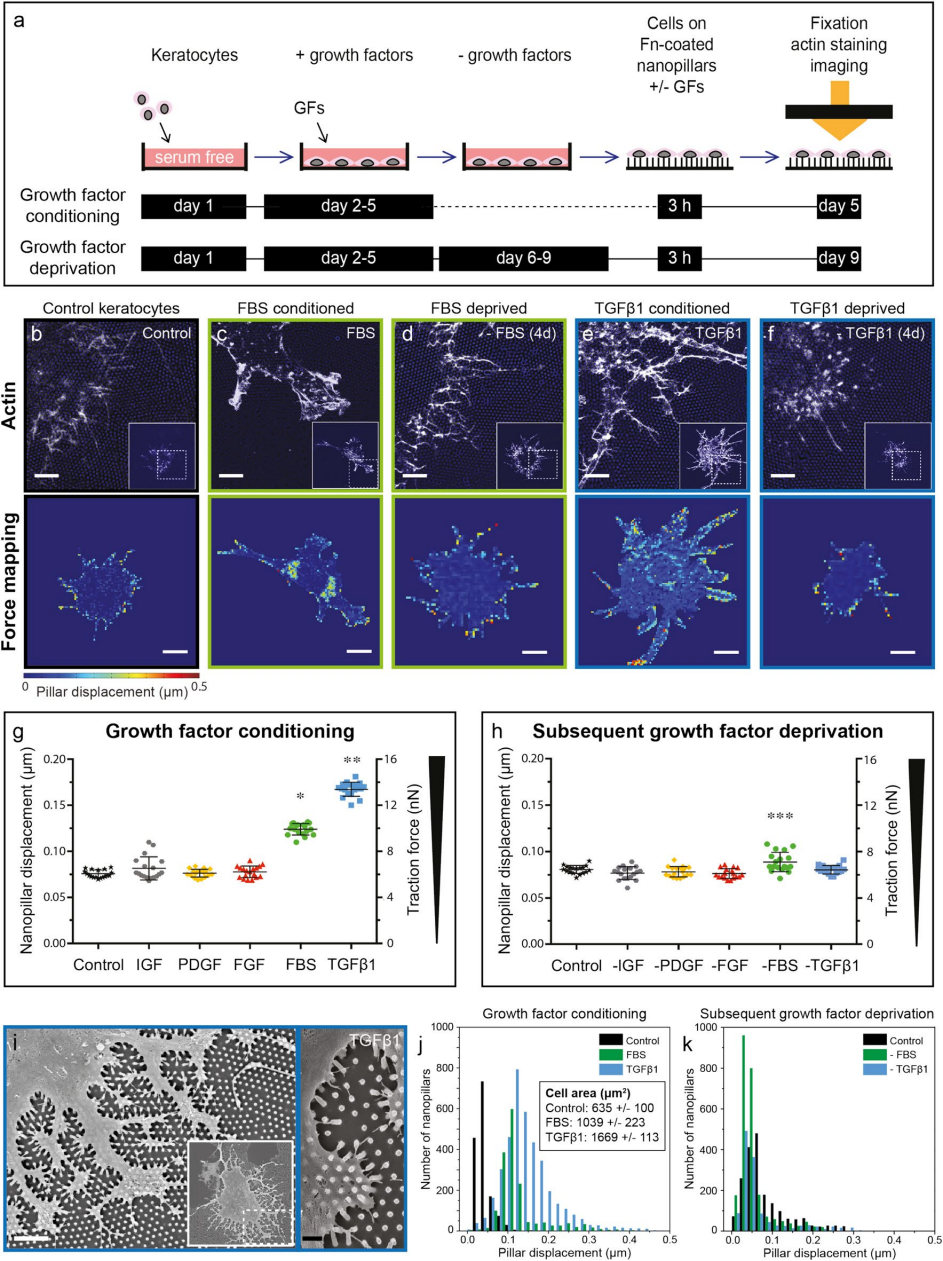
TGFβ1 and FBS conditioning cause an increase in cell contractility and redistribution of cellular forces, which is reversed via TGFβ1 and FBS deprivation. (**a**) Experimental timeline: growth factor conditioned and deprived keratocytes were seeded onto Fn-coated nanopillar substrates, fixated three hours after seeding, then phalloidin stained and imaged. Confocal microscopic images were obtained to evaluate the deflection of the nanopillars underneath the cells by recording images at the pillar’s base and top and by analyzing pillar displacement with particle tracking software. (**b**–**f**) Actin cytoskeleton morphology of control, FBS conditioned, FBS deprived, TGFβ1 conditioned and TGFβ1 deprived keratocytes seeded on nanopillar substrates. Scale bars: 5 μm. Corresponding colorimetric distribution maps of force induced pillar displacement across the cell body are juxta-positioned. Scale bars: 20 μm. Displacement of single nanopillars was topographically mapped with the level of displacement indicated by colors ranging from blue to red (0 to 0.5 μm displacement). (**g**) Scatter plot of nanopillar displacement and traction forces per nanopillar generated by growth factor conditioned keratocytes. Displacement of and forces exerted on single nanopillars were averaged across whole cells. FBS (*:*p* < 0.0001) and TGFβ1 (**:*p* < 0.0001) conditioning significantly increased cellular contractility compared to other phenotypes. Plots were constructed from data averaged from 18 cells per phenotype measured in three separate experiments. Bars signify the means and whiskers the standard deviation from the mean, reflecting force differences between individual cells. Statistical comparisons via one-way ANOVA and Tukey’s multiple comparisons test with significance set at p < 0.05 for all comparisons. (**h**) As (**g**), but for growth factor deprived keratocytes. Note residual increased contractility of FBS deprived keratocytes (***:*p* < 0.01–0.0001). (**i**) SEM images of TGFβ1 conditioned keratocyte on nanopillar substrate, right panel illustrating pillar deformation. White scale bar: 5 μm, black scale bar: 1 μm. (**j**, **k**) Histograms of pillar displacement in μm (x-axis) below three individual cells: control, FBS and TGFβ1 conditioned (**j**), and deprived (**k**) keratocytes. Cell size (average and standard deviation) was calculated based on the number of nanopillars covered by the cells (area under the curve) for 10 cells per phenotype.

A remodeling of the contractile actomyosin cytoskeleton is crucial for cell phenotype transitions (Supplementary Figs. S1 and S5)^28,35,36^. To quantify how this relates to changes in cell contractility during the K–F/M transition and reversal, Fn coated SU-8 nanopillar arrays were used to directly measure cellular contractility on a single cell level in our K–F/M transition model (Fig. 2a)^30^. Quantification of the mean forces per nanopillar exerted by IGF-1, PDGF or FGF conditioned keratocytes revealed that they did not exceed the native keratocyte baseline (Fig. 2g). In contrast, FBS conditioned fibroblasts generated significantly higher traction forces on the nanopillar substrates compared to native keratocytes (Fig. 2c,g,j). Forces generated by TGFβ1 conditioned myofibroblasts were significantly higher than for any of the other phenotypes tested (Fig. 2e,g,j). FBS and TGFβ1 conditioned keratocytes not only generated higher traction forces per nanopillar (Fig. 2g,j), but also increased significantly in cell size (Fig. 2j, see also^33^), resulting in much higher overall force generation per cell. In parallel with their increased contractility, actin fiber assembly was significantly upregulated in FBS and TGFβ1 conditioned keratocytes on Fn-coated nanopillar substrates, but not with IGF-1, PDGF or FGF conditioning (Fig. 2b,c,e, Supplementary Fig. S6).

Growth factor conditioning not only had a major impact on cell shape, but also on the locations of maximal pillar displacements (Fig. 2b–f, supplementary Fig. S6). While the traction forces were highest at the cell periphery for keratocytes, IGF and PDGF conditioned cells, FBS or TGFβ1-activation led to the additional displacement of perinuclear pillars (Fig. 2b,c,e, Supplementary Figs. S6, S7). We previously demonstrated that the high perinuclear pillar displacement originates from actin stress fibers mostly oriented along the main cell axis and spanning the apical side of the cell nucleus, also called the ‘actin cap’^30^.

Actin stress fibers were only observed in FBS conditioned keratocytes on nanopillars, but not upon TGFβ1 stimulation in our study (Supplementary Fig. S8). Instead, a dendritic cell shape resembling the myofibroblast morphology in compliant collagen gels, and a dense actin network, rich in nodular actin structures colocalized with regions of high pillar displacement, were observed within TGFβ1 conditioned keratocytes on nanopillars (Figs. 2e,i, Supplementary Figs. S7–S9)^37^.

**Figure 3:**
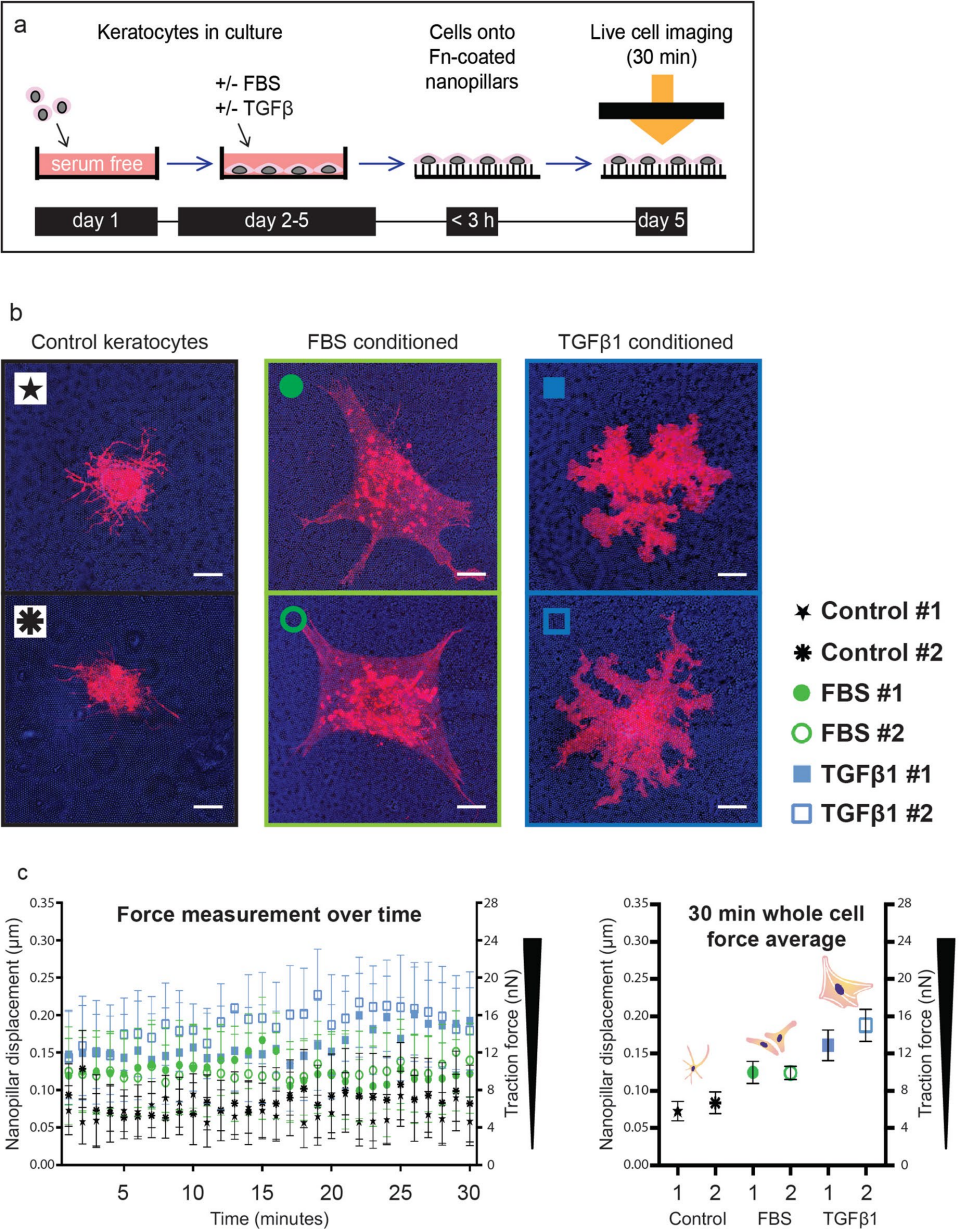
Dynamic traction force measurements in native, FBS and TGFβ1 conditioned keratocytes reveal contractile forces that are stably maintained over a 30-min timeframe. Force generation measurements on fixed cells reflect the cellular contractile state at a single time point, which was three hours after cell seeding in our single cell contractility assays (Fig. 2a). Cellular force generation changes during the early cell attachment and spreading phases^38^. We therefore asked whether significant changes in cellular force generation occurred in our model between two and three hours after cell seeding. (**a**) Native, FBS and TGFβ1 conditioned keratocytes were live-membrane (DiL) stained and seeded onto Fn-coated nanopillar substrates. Two cells per phenotype were imaged within three hours after seeding for a period of 30 minutes on an incubated microscope stage. This timeframe was chosen to match the timeframe for force generation measurements on fixed cells and evaluate traction force stability after the initial cell attachment and spreading phases^38^. Nanopillar displacement was evaluated using particle tracking software on confocal microscopic images. (**b**) Cell outlines of DiL-stained native, FBS and TGFβ1 conditioned keratocytes seeded on nanopillar substrates. Scale bars: 10 μm. (**c**) Plots of displacement of and forces exerted onto single nanopillars averaged across whole cells. Minute-by-minute force plots (left graph) and 30-min whole cell force averages (right graph: two cells per phenotype indicated on the x-axis). The symbols indicate the means and the whiskers the standard deviation from the mean, reflecting force differences between individual nanopillars (left graph) and force differences over time (right graph). Force differences between individual nanopillars are large, as also seen on the pillar displacement histograms in Fig. 2j and the force maps in Fig. 2b–f, where areas with high pillar deflection and areas without pillar deflection are identified in all three phenotypes. The traction forces (nN) observed during dynamic force measurement resembled the forces measured in experiments with fixed cells, with the highest forces measured in myofibroblasts. Nanopillar displacement ranged between 0.05 and 0.10 μm for keratocytes, between 0.10 and 0.15 μm for fibroblasts and between 0.15 and 0.20 μm for myofibroblasts both in live and fixed conditions (Figs. 2g and 3c).

Since cellular force generation changes during the early cell attachment and spreading phases^38^, we asked whether significant changes in cellular force generation occurred in our model between two and three hours after cell seeding. The traction forces obtained during dynamic force measurements on nanopillar arrays (Fig. 3c) were identical to the forces measured in experiments with fixed cells (Fig. 2g), with the highest forces measured in myofibroblasts.

Subsequently, we evaluated whether growth factor stimulated phenotype changes could be reversed by growth factor deprivation in 2D in vitro cell culture conditions. A disassembly of actin stress fibers and a partial reversion towards the native stellate keratocyte morphology was indeed observed following a 4-day period of PDGF or FGF deprivation on planar substrates (Supplementary Fig. S1). Significant actin stress fiber disassembly also occurred in FBS and TGFβ1 deprived keratocytes, but cell polarization and thin basal stress fibers remained visible in most cells cultured on planar substrates (Supplementary Fig. S1). αSMA containing stress fiber disassembly was complete after FBS and TGFβ1 deprivation and cell metabolic activity decreased to the pre-growth factor conditioning levels of native keratocytes following growth factor deprivation (Supplementary Fig. S4).

The generated traction forces, cytoskeletal F-actin morphology, and location of maximal pillar displacement by activated fibroblasts and myofibroblasts (FBS or TGFβ1-treated) on nanopillar array substrates reverted to those of native keratocytes following growth factor deprivation (Fig. 2d,f,h,k).

### FBS and TGFβ1 conditioned keratocytes cause significant stretching of Fn fibers

**Figure 4:**
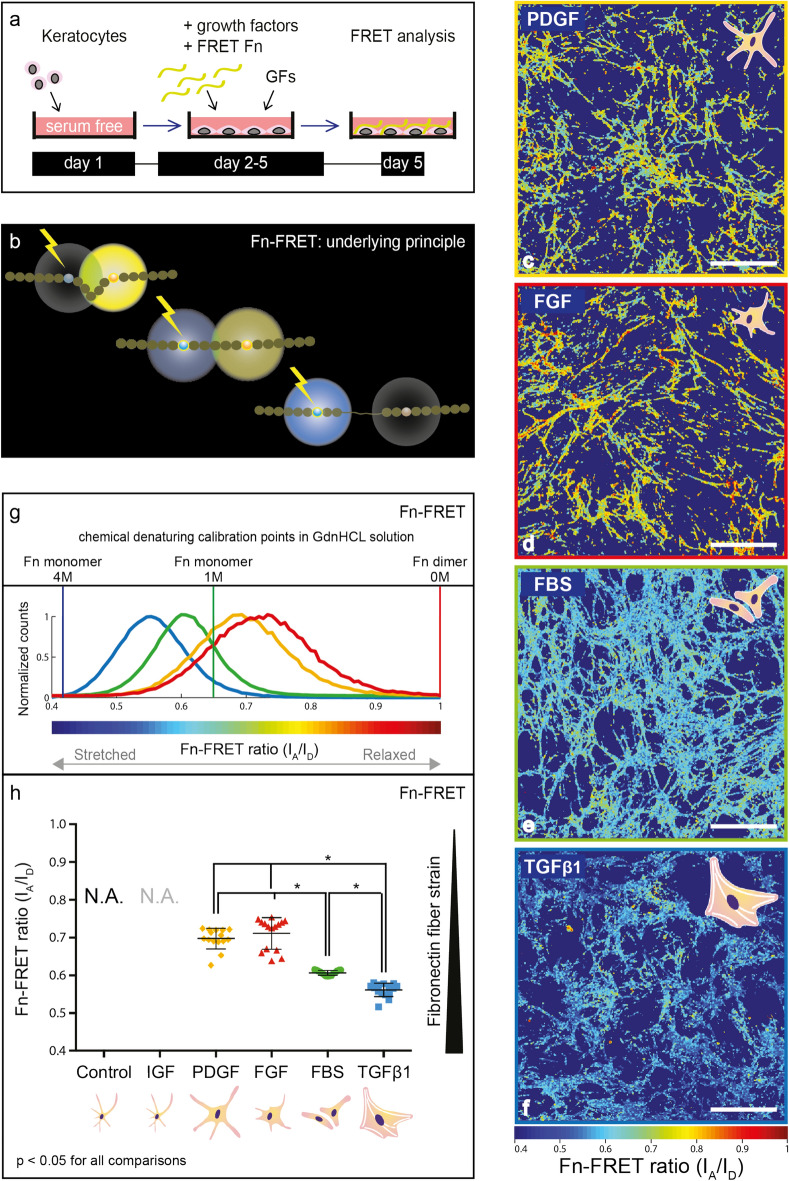
Upregulated keratocyte contractility leads to enhanced fibronectin matrix fiber stretching as probed by FRET. (**a**) Experimental timeline: as described in Fig. 1, but with the addition of FRET Fn—10% Amine/cys double, donor–acceptor fluorophore labeled Fn and 90% unlabeled Fn—to the culture medium to be incorporated into the ECM assembled during the growth factor conditioning period^19,31^. (**b**) Principle of Fluorescence Resonance Energy Transfer (FRET). Multiple donor and acceptor fluorophores label single Fn fibers to probe a large range of conformational Fn changes (loss of tertiary and secondary protein structure folding) using well established protocols. Energy transfer efficiency between donor and acceptor fluorophores decreases as the donor–acceptor distance increases during Fn protein unfolding, measured as a decrease in Fn-FRET ratio: acceptor (I_A_) divided by donor (I_D_) channel fluorescence emission intensity. ECM assembly by native control keratocytes or IGF-1 conditioned keratocytes was not detectable in this assay. A Fn-FRET ratio decrease is visualized as blue shift in the color-coded I_A_/I_D_ ratiometric images for PDGF (**c**), FGF (**d**), FBS (**e**) and TGFβ1 (**f**) conditioned keratocytes, and as left-shift in the corresponding Fn-FRET histograms: PDGF (orange), FGF (red), FBS (green) and TGFβ1 (blue) (**g**). Histograms were derived from one representative field of view from one of three separate experiments in each group. FRET ratios for Fn in solution under denaturing conditions in the presence of GdnHCL [dimeric Fn-DA in 0 M GdnHCL (1) and monomeric Fn-DA in 1 M (0.65) and 4 M GdnHCl (0.42)] are shown as vertical red, green, and blue lines, respectively. (**h**) Significant differences in Fn molecule unfolding were observed between ECM samples derived from TGFβ1, FBS, FGF and PDGF conditioned keratocytes. Scatter plots were constructed from data averaged from five random fields of view each from three separate experiments per group. Bars signify the means and whiskers the standard deviation from the mean. Statistical comparisons: one-way ANOVA and Tukey’s multiple comparisons tests, significance set at *p* < 0.05 for all comparisons. Scale bars: 50 μm.

To ask how differences in Fn and collagen deposition and cellular contractility between FGF, PDGF, FBS or TGFβ1 conditioned keratocytes impact Fn fiber tension, our well validated Fluorescence Resonance Energy Transfer (FRET) assay using Fn labeled with multiple donor and acceptor fluorophores (FRET-Fn) was exploited. This is a sensitive method to probe a large range of conformational changes in Fn fibers^31^. To prevent intermolecular FRET, the FRET-Fn probe was added in trace-amounts to the culture medium (Fig. 4a) using previously published protocols^31^. FRET revealed that FBS and especially TGFβ1 conditioned keratocytes stretched the Fn fibers within the ECM much more than PDGF and FGF conditioned keratocytes (Fig. 4c–h). We know from previous studies that cell-regulated Fn fiber stretching is associated with partial protein unfolding which can activate or destroy molecular binding epitopes^19^. Significant Fn fiber stretching and thus partial protein unfolding was observed in the ECM assembled by FBS and especially TGFβ1 conditioned keratocytes. This finding is in agreement with the enhanced cell contractility of FBS and TGFβ1 conditioned keratocytes as quantified on nanopillar substrates (Fig. 2g). In fact, RhoA activation has been shown to upregulate cell traction forces, as well as Fn fiber tension^19^.

When asking whether the tensional state of the Fn ECM could be reverted, a 4-day period of PDGF and FGF deprivation, subsequent to the initial PDGF and FGF stimulation, revealed a near complete disassembly of the Fn ECM. A significantly decreased Fn ECM density precluded a reliable evaluation of FRET intensity ratios after FBS and TGFβ1 deprivation in vitro. As a result, we could not analyze the Fn fiber tension in growth factor deprived keratocyte cultures. Intracellular Fn-containing vesicles with a fluorescence at the wavelength range of donor fluorophores were observed within TGFβ1-deprived cells (Supplementary Fig. S10), which is suggestive of Fn fiber degradation and internalization^39^.

Inhibition of cell generated forces via latrunculin-B-induced actin cytoskeleton disruption led to partial refolding of Fn as probed by FRET within matrix fibrils in experiments conducted with human foreskin fibroblast (HFF) assembled ECM (Supplementary Fig. S11). However, the myofibroblast-assembled ECM maintained a higher Fn fiber strain than the fibroblast-assembled ECM upon latrunculin-B-induced force inhibition, demonstrating that a residual strain was preserved within the matrix, perhaps due to enhanced ECM fiber crosslinking (Supplementary Fig. S11). This could be due to the ECM crosslinking enzyme transglutaminase 2 (TG2), as TGFβ1 is a direct stimulator of the transcription of TG2^40^. TG2 has various targets amongst ECM fibrils, including Fn, fibrillin and several collagen types, and can effectively cross-link them, thus stiffening the ECM and protecting the ECM from proteolytic degradation^41^. By virtue of its targets, TG2 likely is an important stabilizer of both the early and late wound healing matrix. We thus stained for TG2 and observed that the expression of TG2 was indeed clearly upregulated in TGFβ1 conditioned keratocytes, whereas cells exposed to the other growth factors showed minimal TG2 staining (Supplementary Fig. S12). Matrix crosslinking by TG2 is therefore a likely cause for the observed Fn strain preservation in myofibroblast-deposited ECM in our study^41^. Residual ECM Fn fiber strain was also preserved within the myofibroblast-assembled ECM after matrix decellularization (Supplementary Fig. S11).

### Collagen-dominated and myofibroblast-derived ECM scaffolds reduce K–F/M upon TGFβ activation

We observed that the ECM assembled by various keratocyte phenotypes displayed different Fn fiber tensions (Fig. 4) and collagen content (Fig. 1), and that Fn fiber strain was preserved within the myofibroblast-assembled ECM after matrix decellularization (Supplementary Fig. S11). We therefore asked whether a decellularized fibroblast or myofibroblast derived matrix might constitute a ‘bad neighbourhood’ ECM with a regulatory role in the K–F/M process. We also asked how matrix collagen might impact the niche properties.

**Figure 5:**
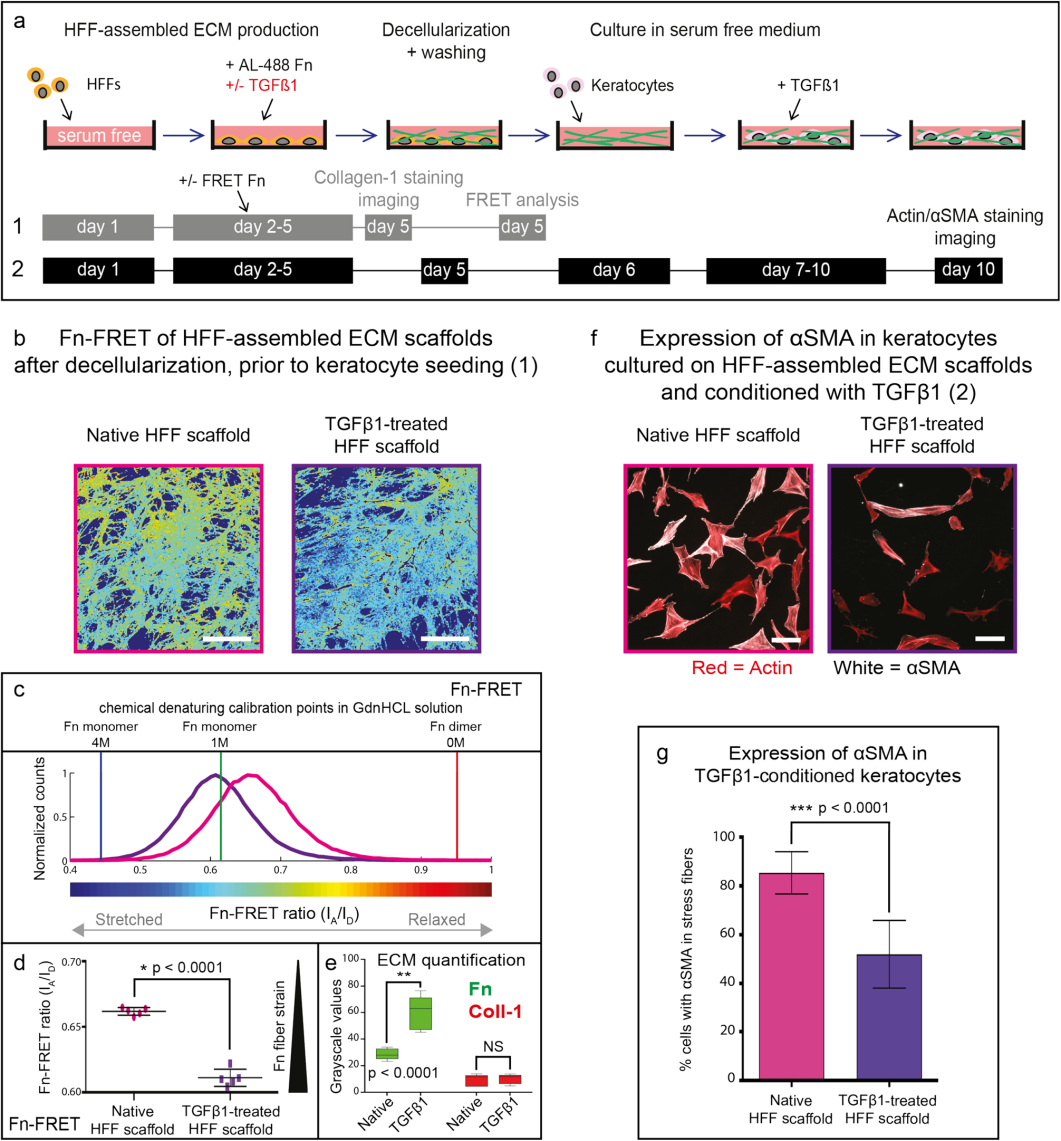
αSMA expression by TGFβ1 conditioned keratocytes is decreased on decellularized myofibroblast-derived ECM scaffolds low in fibrillar collagen-1. (**a**) Experimental timeline: ECM assembled by human foreskin fibroblasts cultured in L-ascorbic acid/Vitamin C-free and serum-free culture medium, with (TGFβ1-treated HFFs) or without (native HFFs) TGFβ1 supplementation for four days. After cell adhesion, medium was replaced with identical medium containing FRET-Fn (see Fig. 4a) or 5 µg/ml Alexa-488 labeled Fn + 45 µg/ml unlabeled exogenous plasma Fn. ECM quantification was performed before and FRET analysis after decellularization. Keratocytes were seeded onto the remaining decellularized ECM scaffolds and exposed to 5 ng/ml TGFβ1 for 4-days. αSMA-incorporation into actin stress fibers was compared in TGFβ1 conditioned keratocytes cultured on native HFF and TGFβ1-treated HFF-assembled ECM scaffolds. (**b**–**d**) FRET evaluation prior to keratocyte seeding. (**b**) Color-coded IA/ID ratiometric images for native (violet) and TGFβ1-treated (purple) HFF-derived scaffolds. Scale bars: 50 μm. (**c**) Histograms of donor–acceptor intensity ratio distributions from the same ECM scaffolds. Histograms were derived from one representative field of view from one experiment in each group. Solution denaturation values for dimeric Fn-DA: 0 M GdnHCl (0.95, red), and monomeric Fn-DA: 1 M (0.62, green), 4 M GdnHCl (0.44, blue). (**d**) Unfolding of Fn within matrix fibrils was significantly greater in ECM scaffolds derived from TGFβ1-treated (purple) HFFs, compared to native (violet) HFFs (*:*p* < 0.0001). Scatter plots were constructed from data averaged from five random fields of view from one experiment comparing both groups. (**e**) ECM quantification was performed as described for Fig. 1c. TGFβ1 supplementation significantly increased Fn (**:*p* < 0.0001), but not collagen assembly. (**f**, **g**) αSMA incorporation into actin stress fibers was observed in 85% of TGFβ1 conditioned keratocytes on native HFF-assembled ECM scaffolds (violet), compared to 52% on TGFβ1-treated HFF-assembled ECM scaffolds (purple) (***:*p* < 0.0001). Scale bars: 100 μm. Measurements from > 200 cells per scaffold type were included in the bar chart: bars signify the means and whiskers the standard deviation from the mean. Statistical comparisons via one-way ANOVA with Sidak’s multiple comparisons test (Fn/Collagen-1) and unpaired t-tests (αSMA), significance set at *p* < 0.05 for all comparisons.

**Figure 6:**
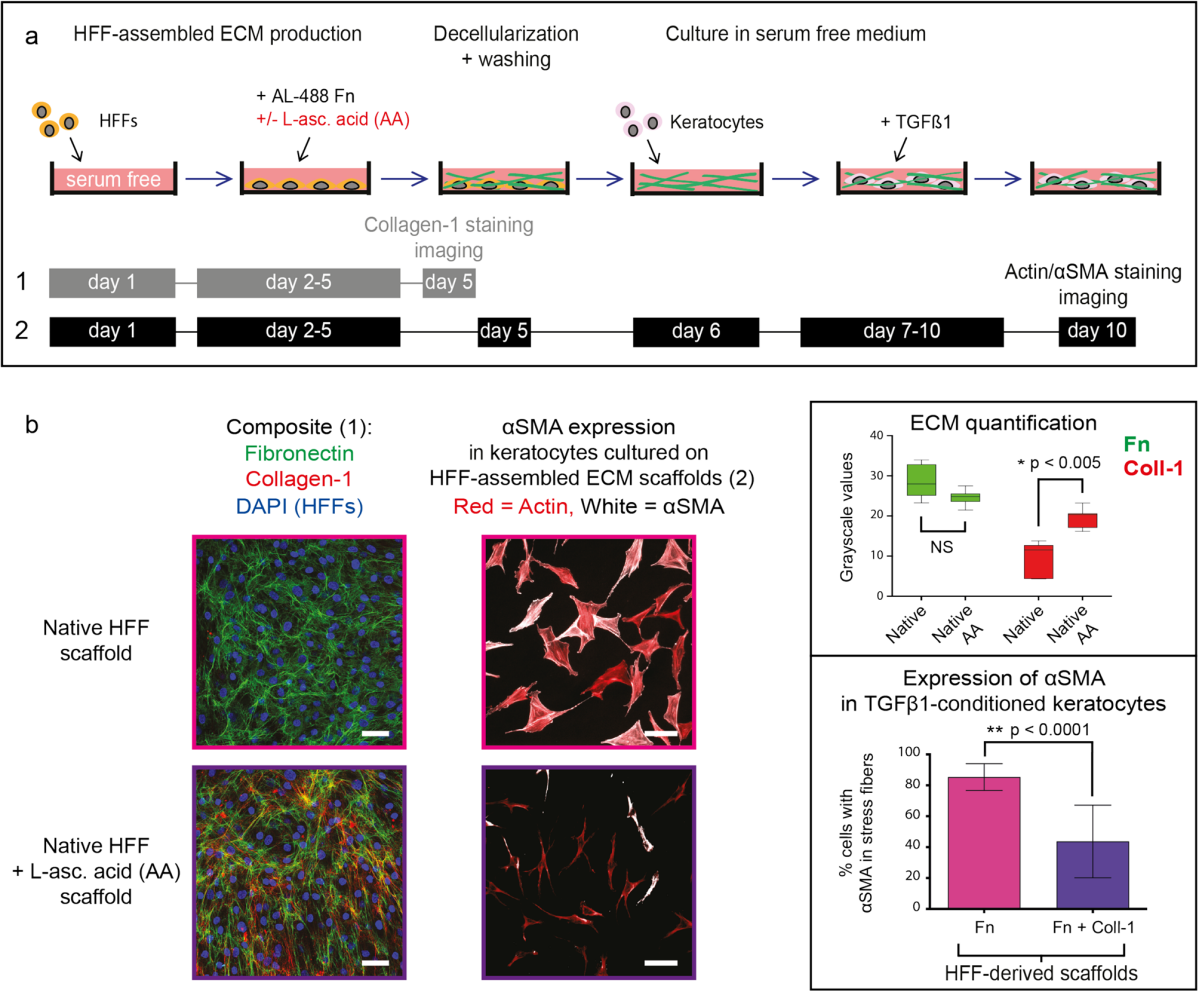
Keratocytes seeded onto decellularized collagen-1 fiber-rich ECM scaffolds show downregulated αSMA expression, despite TGFβ1 stimulation. (**a**) Experimental timeline: human foreskin fibroblasts were cultured in serum-free culture medium with (Fn + Coll-1) or without (Fn) L-ascorbic acid added to allow a 4-day period of ECM assembly. A subset of ECM scaffolds was imaged after Collagen-1 immunostaining. The rest of the scaffolds underwent decellularization and further processing as detailed in Fig. 5a. (**b**) Left column and box plot: native (violet) and L-ascorbic acid supplemented (purple) HFFs both assembled a Fn-rich ECM on account of the supplied plasma Fn in the culture medium. ECM quantification was performed as described for Fig. 1c. L-ascorbic acid supplementation did not further increase Fn assembly, but significantly increased collagen assembly (*:*p* < 0.005). Scale bars: 50 μm. Middle column and bar chart: αSMA incorporation into stress fibers was observed in 85% of TGFβ1 conditioned keratocytes on native HFF-assembled ECM scaffolds (violet: Fn), compared to 44% on L-ascorbic acid supplemented HFF- assembled ECM scaffolds (purple: Fn + Coll-1) (**:*p* < 0.0001). Scale bars: 100 μm. Measurements from > 200 cells per scaffold type were included in the bar chart: bars signify the means and whiskers the standard deviation from the mean. Statistical comparisons were performed via one-way ANOVA with Sidak’s multiple comparisons test (Fn/Collagen-1) and unpaired t-tests (αSMA), with significance set at *p* < 0.05 for all comparisons.

In these experiments, HFFs were cultured in the presence or absence of TGFβ1 and/or L-ascorbic acid (Vitamin C) during ECM assembly. The Fn and collagen content were quantified in a subset of the assembled scaffolds. Supplementation of TGFβ1 significantly increased Fn assembly (Fig. 5e, Supplementary Fig. S13) and Fn stretching within matrix fibers in the assembled ECM scaffolds (Fig. 5b–d), similar to the situation in the ECM assembled by growth factor-treated keratocytes (Figs. 1, 4). Collagen fibril deposition was significantly increased following L-ascorbic acid supplementation (Fig. 6b).

In the first experiment, low-collagen ECM scaffolds assembled by native HFFs were compared to low-collagen ECM scaffolds assembled by TGFβ1-treated HFFs (Fig. 5a, Supplementary Fig. S13). To investigate ECM scaffolds with low collagen content, the first experiment was run without L-ascorbic acid supplementation, but with the supplementation of FRET-Fn during the 4-day culture period prior to decellularization. ECM quantification before and FRET analysis after decellularization confirmed that the ECM assembled by TGFβ1-treated HFFs contained much more stretched Fn within matrix fibers compared to native HFF derived ECM (Fig. 5b–e). In the second experiment, collagen-1-rich ECM scaffolds (L-ascorbic acid supplementation during ECM assembly) were compared to low-collagen-1 ECM scaffolds (L-ascorbic acid deprivation during ECM assembly), all assembled by native HFFs (Fig. 6). In the third experiment, low-collagen ECM scaffolds assembled by L-ascorbic acid-deprived native HFFs were compared to collagen-1-rich ECM scaffolds assembled by L-ascorbic acid and TGFβ1-supplemented HFFs (Supplementary Fig. S14). After scaffold decellularization, keratocytes were seeded onto the ECM scaffolds and 5 ng/ml TGFβ1 was added for four days to stimulate myofibroblast differentiation. Importantly, the proportion of TGFβ1 conditioned keratocytes immunocytologically expressing αSMA-positive actin stress fibers was significantly lower on TGFβ1-treated HFF-derived scaffolds and on collagen-1-rich scaffolds (Figs. 5f,g, 6b, Supplementary Fig. S14).

## Discussion

As the cornea is a living material that needs to stay transparent under healthy conditions, yet needs to be repairable following injury, the challenge nature had to solve is how to close a corneal wound site, and subsequently remodel the altered ECM to restore transparency. Improper healing of the cornea after injury or infections can lead to corneal fibrosis, which causes enhanced light scattering, resulting in vision impairment or even vision loss^1,42–44^. We thus asked how the up- and downregulation of growth factors, simulated by external IGF-1, PDGF, FGF, FBS or TGFβ1 supplementation and withdrawal, affects the keratocyte’s ability to assemble and model the surrounding ECM. And how biophysical alterations of the cellular environment, in concert with growth factor availability, can coregulate a reversible cell phenotype switch.

**Figure 7:**
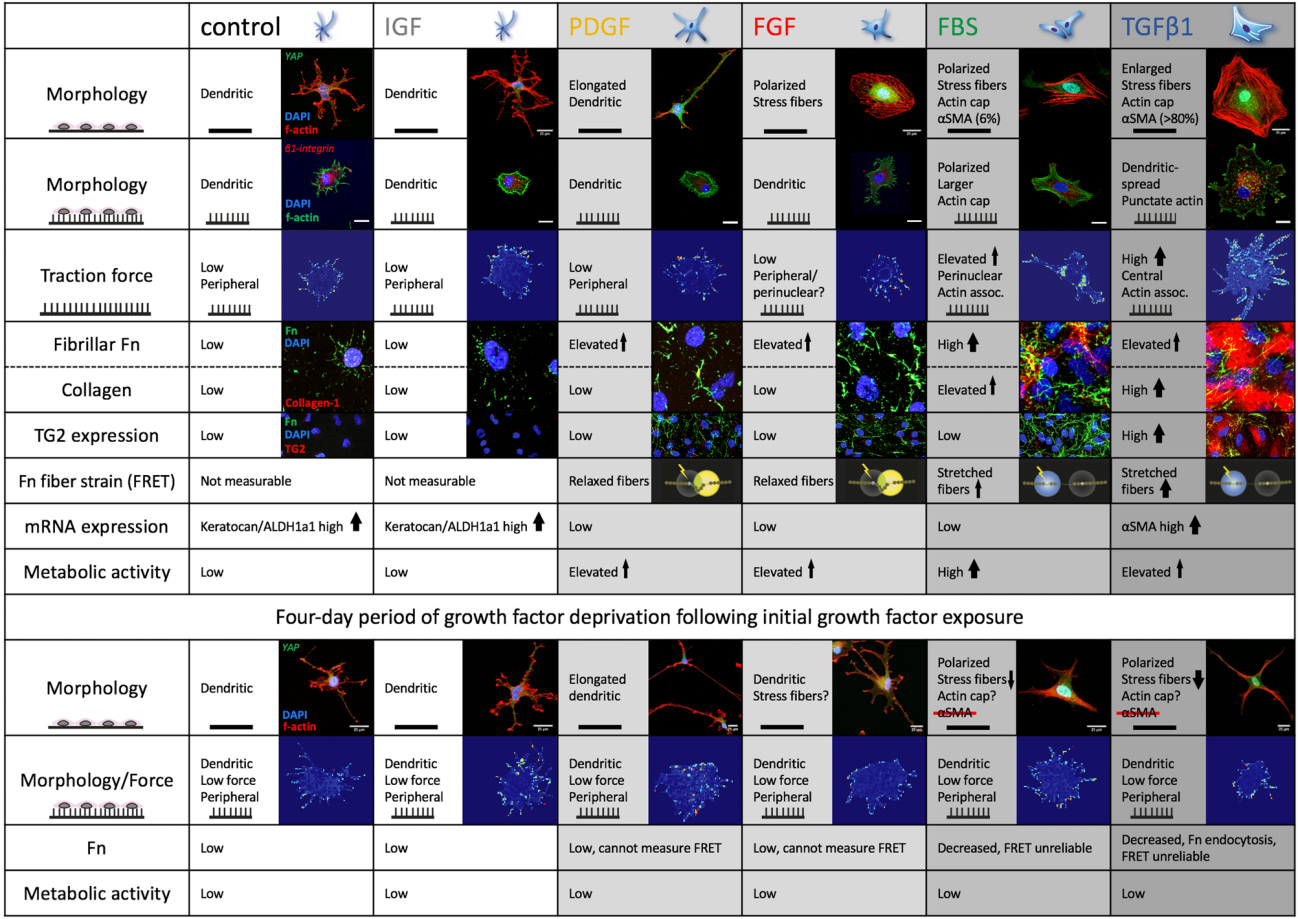
Summary of growth factor induced cell morphologies and behaviors.

We found that each of these wound healing-associated growth factors induced a distinct set of cell morphologies and behaviors as summarized in Fig. 7.

In contrast to growth factors upregulated in wound sites, we did not observe differences between IGF-1-treated and control keratocytes (Fig. 7), which fits the role of IGF-1 in the maintenance and repair of the normal corneal keratocyte network and ECM^5,27,32^.

**Figure 8:**
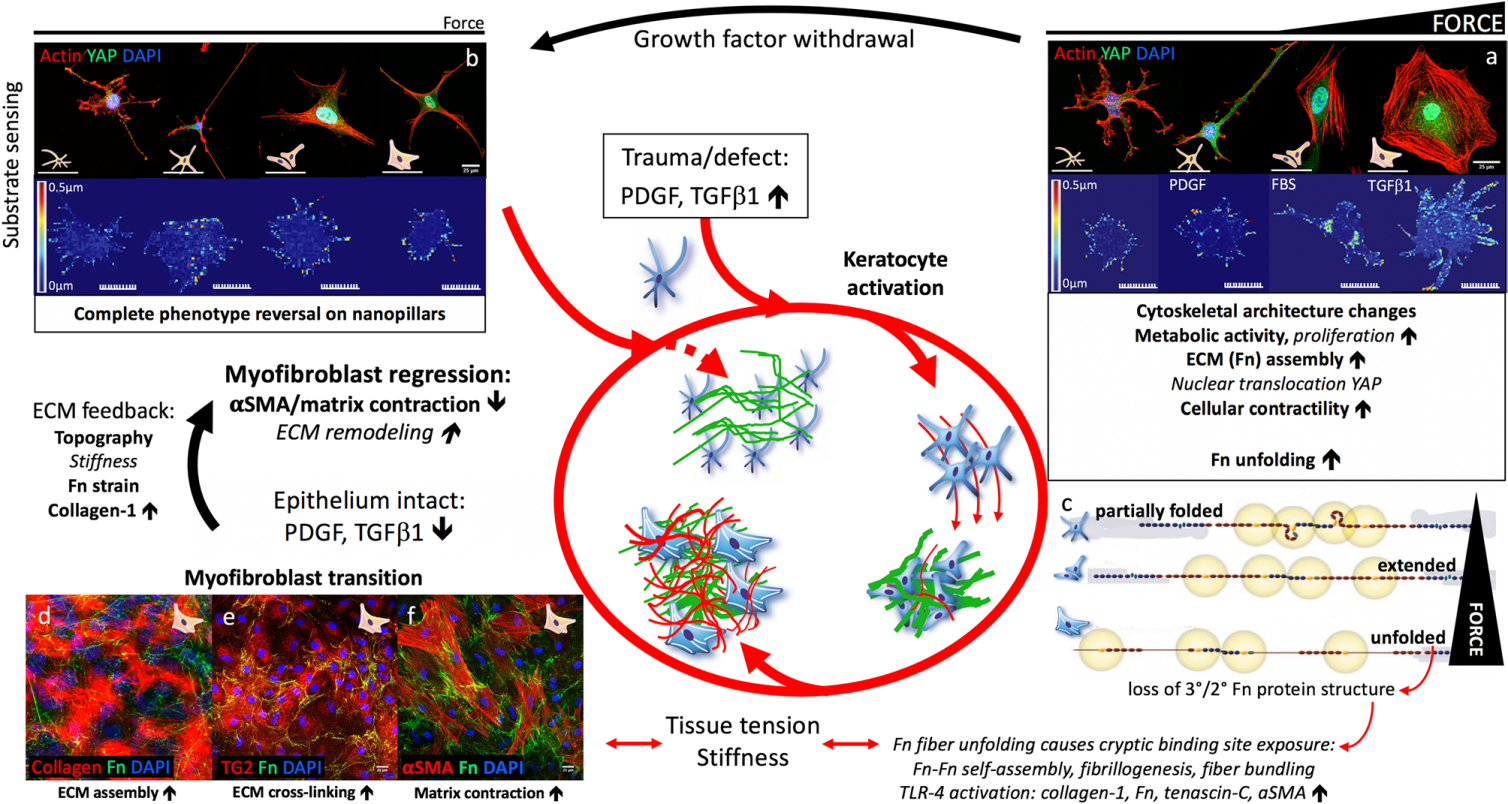
Summary corneal fibrosis and remodeling feedback. Corneal epithelial damage-induced release of soluble factors, notably PDGF and TGFβ1, leads to keratocyte activation. (**a**) Activated keratocytes (from left to right: control keratocyte, PDGF-primed fibroblast, FBS-primed fibroblast, TGFβ1-primed myofibroblast) demonstrate cell morphology changes, increased contractility, metabolic activity, Fn matrix assembly and Fn fiber unfolding. (**b**) Growth factor withdrawal from cell culture mimicked the decrease in stromal growth factor levels after epithelial and basement membrane healing. Substrate type contributed to the completeness of cell phenotype reversal (compare **b** to **a**), meaning that substrate sensing by cells contributes to their phenotype development. (**c**) Fluorescence Resonance Energy Transfer (FRET) experiments revealed that cell generated forces are translated into mechanical ECM strain (Fn unfolding). The contractile phenotypes -FBS and TGFβ1 primed (myo)fibroblasts- stretched Fn fibers within the ECM to a level where partial secondary/tertiary Fn protein structure loss was present. Figure adapted from: Smith et al., Plos Biol, 2007, with permission. Such Fn fiber unfolding exposes cryptic Fn-Fn self-assembly sites^19,22^, accelerating Fn fibrillogenesis, cross-linking and fiber bundling, and stabilizing the early Fn matrix^19,22,23^. Fn fiber unfolding also exposes a cryptic Toll-like receptor (TLR) 4 activating site on Fn’s ED-A domain, resulting in TLR activation, and subsequent TGFβ, tenascin-C (TNC), Fn, and collagen-1 gene expression^48^. Cell generated force-induced Fn fiber stretching can thus promote early Fn fibrillogenesis^22^, and the subsequent assembly of a collagen matrix^48^. Increased solid tissue stresses and tissue tension, caused by cell proliferation, contractility, and matrix assembly, can also drive profibrotic gene expression (incl. αSMA, collagen, TNC), and thus myofibroblast transition and the deposition of a contracted, dense, crosslinked collagen-1-rich matrix (**d**–**f**), in a self-amplifying process^49^. Finally, decreasing PDGF and TGFβ1 concentrations following epithelial and basement membrane healing, together with normalizing ECM properties, including tissue specific matrix topography, stiffness, and collagen fiber content, likely facilitate the disappearance of myofibroblasts from wound sites. Thus, an ECM niche supportive of homeostasis and regenerative remodeling is created. We have integrated our experimental results (bold) with published information in the literature (italics): for citations, please see main text.

Epithelial damage increases stromal levels of PDGF^6^, and PDGF-primed corneal keratocytes are a proliferative, low contractile, metabolically active cell phenotype, displaying collective, contact-guided migration, and the assembly of a relaxed, fibrillar Fn matrix along their migration tracks^2,45,46^ (Fig. 7). PDGF-primed corneal keratocytes therefore seem ideally suited to initiate wound repair by repopulating low stiffness stromal wound areas after keratocyte apoptosis, without causing major matrix remodeling^46,47^ (Fig. 8).

In contrast to the low contractile PDGF-primed phenotype, FBS and TGFβ1-conditioned keratocytes deposited the densest Fn and collagen matrix, and caused significant contraction, fibronectin fiber stretching and reorganization within their surrounding matrix^2,5^ (Fig. 7). These observations are in agreement with previous reports of accelerated Fn fibril formation and matrix assembly as a result of Fn fiber stretching and the exposure of cryptic Fn-Fn self-assembly sites^19,22^. Fn fiber unfolding is also speculated to expose the cryptic Toll-like receptor (TLR) 4 activating site on Fn’s ED-A domain, a Fn splice variant associated with fibrosis^19,48^. Subsequent TLR activation drives the transitioning towards myofibroblasts which show upregulated TGFβ, tenascin-C, Fn and collagen-1 gene expression, and consequently enhanced ECM assembly^48^. Cell generated force-induced Fn fiber stretching can thus promote early Fn fibrillogenesis^22^, but also the subsequent assembly of a collagen matrix^48^. The decreased enzymatic digestion of collagen fibers under tension^50^ further highlights the importance of cell contractility for matrix assembly and stabilization. Vice versa, mechanobiological cues, including highly stretched Fn fibers, were identified as drivers and stabilizers of the myofibroblast phenotype in the growth front of de novo grown microtissues^35,51^.

Serum protein-primed (here: FBS), highly metabolically active, proliferative, contractile and Fn assembling fibroblasts could thus be responsible for increased fibrotic remodeling of the corneal stroma in areas with significant inflammation-associated blood vessel ingrowth (Fig. 8). Finally, myofibroblasts would be responsible for the deposition of a contracted, dense, crosslinked collagen-1-rich matrix (Fig. 8). While these processes are essential for the closure of corneal laceration or perforation wounds, they can create adverse effects in terms of ECM architecture and transparency in situations where cell repopulation, but not wound closure, is needed (e.g. refractive surgery, corneal cross-linking).

Since keratocytes in their native niche are exposed to a nanostructured rather than smooth/planar microenvironment, it is particularly notable that FGF and TGFβ1 primed keratocytes in 2D cell culture adopted a markedly different morphology on nanopillar substrates compared to planar glass in our study. Whereas FGF primed keratocytes maintained the dendritic morphology of control keratocytes on nanopillar arrays, TGFβ1 priming induced an enlarged, dendritic cell shape with a dense actin network, accentuated by nodular actin condensations (Fig. 7, Supplementary Figs. S6–S9). Depending on pillar aspect ratio, nanopillar substrates can be perceived as soft due to cell force-induced pillar bending^52^. Therefore, one explanation for the observed cell morphology difference between planar and nanopillar substrates is that the contractile cells can displace the nanopillars upon force generation, thus sensing a ‘soft’ substrate. Whereas their adhesion sites are not displaced on planar substrates of equally rigid material, causing the cells to indeed perceive such surfaces as rigid. In support of this notion, similar behaviour was observed in previous work demonstrating a preservation of the dendritic morphology by FGF-primed keratocytes^53^, and of the enlarged, dendritic cell morphology by TGFβ1-primed keratocytes^37^, in uncompressed soft 3D collagen gels. On the other hand, compressed stiff 3D gels promoted the classical FGF-induced polarization with presence of actin stress fibers^53^, and enlarged, stress fiber-rich myofibroblast morphology in TGFβ1-primed keratocytes^37^, like on planar glass in our study (Fig. 7). Furthermore, TGFβ1 conditioned keratocytes cultured on soft or patterned 2D substrates have been shown to reduce αSMA stress fiber expression and contractility compared to cells on rigid or planar 2D substrates^54,55^. FGF and TGFβ1 thus clearly elicit a conditional response, which depends on mechanosensory feedback from the substrate or pericellular matrix to the cells. This response is likely initiated by differences in topography, ligand availability/density, and/or ‘observed’ viscoelasticity^56,57^. The K–F/M transition reversal following growth factor withdrawal was more complete on nanopillar arrays than on planar glass substrates in our study (Fig. 7). These results underline the importance of substrate mechanosensing, and of a normalization of stromal growth factor concentrations following epithelial wound healing^6,7^, for myofibroblast phenotype reversal.

The presence of scar tissue deposited by myofibroblasts in vivo has been proposed to create a ‘bad neighbourhood’-type niche in which fibrosis is promoted and perpetuated, also after the matrix depositing cells have disappeared^9^. In our study, we found that the dense collagen matrix with highly stretched Fn fibers deposited by myofibroblasts, was maintained even after cell removal (Figs. 5b–d, 7, Supplementary Fig. S11). Contrary to the abovementioned ‘bad neighbourhood’ hypothesis, we observed a reduction in the transition of keratocytes into αSMA expressing myofibroblasts when cultured on these myofibroblast-derived, collagen and stretched Fn fiber-rich, decellularized ECM scaffolds. This observation even held true in the presence of exogenously supplemented TGFβ1 (Figs. 5, 6, Supplementary Fig. S14).

Combining our 2D and 3D^35,51^ data suggests two things: first, the composition of the ECM overruled soluble factor signaling to exert a defining influence on the (K-)F/M transition in our cell culture models. The biochemically and biophysically complex ECM within these 2D and 3D environments clearly played a more dominant instructive role on myofibroblast transition than exogenously supplemented TGFβ1, in agreement with studies that used decellularized cancer-associated stroma ECM^11^, or decellularized fibroblast-derived microtissues^51^. This is remarkable, since TGFβ1 has historically been viewed as an essential ingredient for myofibroblast transition^37^. Second, scar tissue ECM does not necessarily need to create a ‘bad neighborhood’-type niche in which fibrosis is promoted. Instead, the fibrotic, myofibroblast-derived ECM may contain cues that favor tissue regeneration over sustained fibrotic scarring. Our data (Fig. 6, Supplementary Fig. S14,^35,51^) suggest that a low Fn/collagen ratio inhibits fibroblast to myofibroblast transition.

Regarding the potential clinical relevance, key features of the wound healing and tissue regeneration phases following in vivo laser ablation surgery of the anterior corneal stroma in rabbits^1^ underline a mechanosensory ECM feedback-induced myofibroblast phenotype reversal. Corneal haze, αSMA and Fn expression, and the presence of enlarged, actin stress fiber-rich myofibroblasts peaked at 21 days after laser ablation surgery. Also, collagen-1 expression was elevated at this timepoint, but only few collagen fibers were detected via SHG within a rather featureless stroma^1^. This situation is most reminiscent of the myofibroblast-promoting environment on planar glass substrates (Fig. 7), in stiff 3D collagen gels^37^, and in the 3D microtissue growth front^35,51^. Between days 60 and 180 after laser ablation surgery, the complex collagen fiber topography of the normal corneal stroma reappeared, with corneal haze, Fn, αSMA and the typical myofibroblast morphology progressively disappearing. At the same time, the reorganizing (myo)fibroblasts increasingly expressed bright punctate, actin-rich structures that were associated and aligned with the collagen fibers^1^. This situation is most reminiscent of the myofibroblast-inhibiting environment on nanopillar substrates (Fig. 7), in soft 3D collagen gels^37^, and in the 3D microtissue core region^35,51^. In a similar in vivo laser ablation surgery study in rabbits, the wounded corneal stroma assumed a maximum bulk tissue stiffness at 7 days after laser treatment^58^. Stromal stiffness was still high at 21 days after laser treatment, when αSMA expression was at its maximum. Stromal stiffness progressively decreased at later measurement timepoints at 42, 70 and 400 days after laser treatment, when αSMA expression, histologically graded fibrosis, and clinical corneal haze decreased as well^58^.

An interpretation of the combined results from these in vivo wound healing studies^1,58^, as well as 2D and 3D cell culture models^35,37,51^, thus supports the presence of an ECM niche supportive of homeostasis and regenerative remodeling. This niche is most likely characterized by a normalized tissue physiology-specific matrix topography, stiffness, and collagen fiber content, overruling soluble factor signaling, and facilitating the disappearance of myofibroblasts from wound sites.

In summary, the growth factor deprivation and ECM-driven downregulation of myofibroblast differentiation observed in our study provide a mechanobiological explanation for the disappearance of (myo)fibroblasts during the maturation phase of wound healing (Fig. 8). Such myofibroblast disappearance should facilitate regenerative matrix remodeling at wound sites and in scars^12^. Remodeling processes in the corneal stroma are active for months or years and determine whether opacities persist or regress^44^. Understanding the basic mechanisms governing cell-ECM crosstalk is becoming increasingly important with the vast increase in popularity of novel clinical and tissue engineering tools that mechanically and biochemically modify the cellular microenvironment and potentially affect cell fate. As such, our findings reflect basic, clinically relevant, and potentially targetable mechanisms related to tissue fibrosis.

## Methods

Detailed experimental protocols are available as supplementary information.

### Primary rabbit keratocyte isolation and cell culture

Eyes used for isolation of primary corneal keratocytes were obtained from healthy rabbits at a local abattoir. The keratocytes were isolated and cultured on collagen coated, plasma treated polystyrene culture dishes (tissue culture plastic) prior to passaging onto various specific substrates for the different experiments. Exposure to specific growth factors (IGF-I: 10 ng/ml; PDGF-BB: 50 ng/ml; FGF-2: 10 ng/ml; TGFβ1: 5 ng/ml) or 10% Fetal bovine serum (FBS) supplemented to the serum free medium using previously described methods^59^ took place either while on the initial collagen coated culture dishes during culture expansion, or after passaging onto the final substrate. Trypsin used for passaging keratocytes was neutralized with Soybean Trypsin Inhibitor. The growth factor concentrations represent the lowest concentrations with a maximal effect on cell morphology and F-actin organization. These concentrations were adopted from previous studies^28,59^. For interventional experiments, cells were seeded onto glass cell culture substrates with adsorbed unlabeled Fn.

### Primary human foreskin fibroblast (HFF) cell culture

Primary human foreskin fibroblasts (HFF) were cultured using previously described protocols^60^.

### Immunocytochemistry

Primary antibodies used: mouse monoclonal anti-α-SMA (O.N.5, Cat# ab 18147 [1A4], Abcam, Cambridge, UK); 1:100 dilution in 1% BSA/PBS for 1 h, used as myofibroblast phenotype specific marker; mouse monoclonal anti-transglutaminase 2 antibody (CUB 7402], Abcam); 1:100 dilution in 1% BSA/PBS for 1 h; goat polyclonal anti-Fibronectin, N‐20 (Cat# sc‐6953, Santa Cruz, CA; 1/100 dilution in 3%BSA/PBS prior to cell permeabilization for 1 h); mouse monoclonal anti-Collagen I ([COL-1], Cat# ab 90395, Abcam, Cambridge, UK; 1/200 dilution in culture medium for 1 h at 37 °C).

Secondary antibodies used: goat anti-mouse-Alexa Fluor 555 (Cat# A21424, Life technologies); Donkey anti-mouse Alexa Fluor 488 (Cat# A-21202, Invitrogen); Donkey anti-goat-Alexa Fluor 633 (Cat# A21082, Life Technologies; all at 1:100–1000 dilution in 1–3% BSA/PBS for 30–60 min).

Compartment specific fluorescent dyes used: phalloidin-Alexa Fluor 488 and 568 (1:100–200 dilution in 1 × PBS + /− 1–3% BSA for 2 h) as cellular F-actin and actin stress fiber markers; 4',6-diamidino-2-phenylindole (DAPI) (1:1000 dilution in 1 × PBS for 10–15 min) to counterstain cell nuclei. Routine fixation, permeabilization, blocking and staining protocols were used. All antibodies and compartment specific fluorescent dyes were added after fixation and permeabilization, with the exception of the primary anti-Coll-1 antibody and the primary anti-Fn antibodies, which were added to the live cell culture at 37 °C and after fixation but prior to permeabilization, respectively. Finally, samples were left in 1 × PBS until immunofluorescent imaging with a Zeiss Axiovert 200 M epifluorescent microscope, or Olympus FV-1000 or Leica SP5 confocal microscope.

### Image analysis for ECM quantification

To ensure robust results, quantification of immunofluorescence intensities in Z-stack confocal microscopy data was carried out using a custom-built FIJI macro, which can be accessed on GitHub (https://github.com/BennSynergy/FIJI-macro_zStackQuant.git, DOI: 10.5281/zenodo.7978897)^61^. It was noted that the z-position of z-stack-slices exhibiting maximum fluorescence intensity significantly varied between the collagen-1 and Fn channel in TGFβ1-treated samples (see Supplementary Fig. S15). Consequently, quantification of Fn and collagen-1 fluorescence intensities was performed in a 3-slice substack that surrounded the z-stack-slice with peak fluorescence intensity in the collagen-1 channel. This strategy was adopted since the selection of the maximum z-position from either the collagen-1 or Fn channel for fluorescence intensity quantification did not alter the relative differences observed among the interventional groups.

### MTT assay

An MTT assay was used as an indicator of cell proliferation in growth factor conditioned keratocytes at culture day 5 and in growth factor deprived keratocytes at culture day 9, and was performed according to the manufacturer’s instructions (Cell Proliferation Kit I (MTT), Cat # 11465007001, Roche). Absorbance of the medium was measured with a Tecan M200 plate reader. Absorbance values were normalized to that of the control keratocytes in serum-free medium and reported in Fig. S4.

### Real-time PCR evaluation of keratocyte and myofibroblast markers

Cells were lysed and RNA was isolated (Nucleospin RNA-II, Cat # 740955.50, Macherey Nagel AG, Oensingen, Switzerland), and cDNA was produced (Taqman^®^ Reverse transcription Reagent, Cat# N808-0234, Applied Biosystems) using manufacturers protocols. A spectrophotometer was used to determine RNA yield (Nanodrop; Thermo Scientific, Wilmington). Gene expression was evaluated by real-time PCR using SYBR green reagents (Sensimix SYBR kit from Bioline) and validated real-time PCR primers for rabbit keratocyte and myofibroblast markers (Table 1, supplementary information) as previously published^33^. Relative quantification was performed by the ΔΔCT method with β-actin used as normalizing housekeeping gene.

### Nanopillar array fabrication and cell traction force measurement

Nanopillar platforms were fabricated exploiting nanosphere lithography followed by a molding process using previously described protocols^30^. The photoresist SU-8 nanopillars measured 0.25 μm in diameter and 1.5 μm in height with a 0.8 μm pillar center to pillar center distance. The spring constant of a representative SU-8 nanopillar was measured by Atomic Force Microscopy (AFM) by deflecting single nanopillars with the AFM cantilever, and was used to calculate the cell-generated horizontal traction forces on the pillar substrate. These nanopillars with passivated pillar sides and Fn-coated pillar tops were biocompatible and allowed a natural spreading of cells on top of the nanopillars. Cells were fixed 3 h after seeding, then phalloidin stained and imaged on a Leica SP5 confocal microscope. This timeframe was chosen since we aimed to measure force generation after the initial cell to substrate attachment phase^38^, but prior to any significant ECM deposition. Since force generation measurements on fixed cells reflect the cellular contractile state at a single point in time and cellular force generation changes over time as adhesion complexes mature^38^ we were interested in force generation measurements over time. To visualize cell edges in live-cell imaging experiments the cells were incubated with a fluorescent membrane dye (Vybrant/ Dil, Invitrogen, 1:200 dilution in culture medium) in suspension prior to seeding. Within 3 h after seeding, each cell was imaged for 30 min on an incubated microscope stage.

The pillar displacement underneath the cells in xy direction was quantified by comparing two sets of images with focal planes at the pillar base and top, respectively. Pillar displacement was analyzed with particle tracking software (Diatrack 3.03, Powerful Particle Tracking, Semasopht; and Fiji, plugin, template matching for drift collection) and the traction forces by which the cells displaced the nanopillars calculated.

### Scanning electron microscopy

Cells on nanopillar arrays were imaged using a Zeiss ULTRA 55 Scanning Electron Microscope after fixation, critical point drying, and gold sputter-coating using standard protocols^30^.

### Direct stochastic optical reconstruction microscopy (dSTORM)

For dSTORM imaging, keratocytes preconditioned with 5 ng/ml TGFβ1 for 4 days, were seeded onto Fn-coated coverslips. After fixation, permeabilization, blocking and staining with Alexa Fluor 647 phalloidin, samples were imaged using a home-built set-up for single-molecule localization microscopy, as previously described^62^.

### Fn isolation and labeling

Fn was isolated from human plasma (Zurcher Blutspendedienst SRK, Switzerland) by affinity chromatography as previously described^31^. Double labeling of plasma Fn with Alexa Fluor^®^ 488 as donor on amines and Alexa Fluor^®^ 546 as acceptor on free sulfhydryls was performed as previously described^31^.

### Preparation of cell derived ECM scaffolds

Tissue equivalents, often collagen gels seeded with fibroblasts, were used to investigate ECM stress regulatory principles in most previous studies^2,5,28^. Although collagen fibrils can self-assemble in vitro, their assembly and proper organization in vivo are regulated by many additional binding partners, including cellular fibronectin and integrins^17^. Furthermore, resident tissue fibroblasts control the supramolecular fibril organization within and the three-dimensional structure of the collagen matrix^63^. Cultured human foreskin fibroblast (HFF) assembled ECM scaffolds were therefore used here to provide a more physiologically relevant 3D cell culture environment to evaluate the influence of the ECM on K–F/M transition. HFFs (50,000 cells/cm^2^) were seeded onto Fn coated surfaces and allowed to adhere for 30 min. The culture medium was then replaced by cell-type specific medium containing FRET labeled Fn or Alexa 488 singly labeled Fn. Cells were cultured for 4 days with a medium change after 48 h prior to imaging.

### Image acquisition and analysis for fluorescent resonance energy transfer (FRET)

Fluorescent Resonance Energy Transfer (FRET) analysis was performed as previously described^31^, using Matlab (http://www.mathworks.com/) with a self-programmed script (script available as supplementary information). Using an Olympus FV-1000 scanning laser confocal microscope, all FRET images were acquired from living cell samples, except for the FRET images from HFF derived ECM scaffolds for keratocyte reseeding experiments and for decellularization experiments, which were acquired from decellularized samples. FRET I_A_/I_D_ ratios were calibrated to different Fn conformations in PBS and various strength GdnHCl solutions. Dimeric and fully folded Fn in PBS showed strong energy transfer whereas monomeric and significantly unfolded Fn-FRET in 4 M GdnHCl showed dramatically decreased energy transfer. According to previous studies on Fn conformations in solution^31^, the I_A_/I_D_ value of monomeric Fn-FRET in 1 M GdnHCl will be used to indicate the very first onset of loss of secondary structure.

### Seeding native keratocytes onto HFF-derived ECM scaffolds

Cell-derived ECM scaffolds assembled by fibroblasts (native HFFs) and myofibroblasts (TGFβ1-treated HFFs) were isolated. After 4 days, the cell monolayer was decellularized by a previously described protocol to yield a cell-free cell derived ECM scaffold^64^. Keratocytes were then seeded onto the acellular matrix, and 5 ng/ml TGFβ1 was added to the serum-free keratocyte culture medium after cell attachment to induce myofibroblast differentiation of the keratocytes. Cells were cultured for 4 days, then fixed and stained with phalloidin and αSMA, and imaged with an inverted Zeiss Axiovert 200 M epifluorescence microscope. The proportion of cells expressing αSMA in their stress fibers was calculated and compared between different groups.

### Statistics

The presence of significant differences between any groups was evaluated by unpaired t-tests or one-way ANOVA for parametric data and by Kruskal–Wallis one-way ANOVA for nonparametric data. Post-hoc tests were performed where applicable to identify significant differences between specific groups: Holm-Sidak’s multiple comparison test for αSMA incorporation into stress fibers, Dunn’s multiple comparison test for the MTT, rt-PCR, and keratocyte Fn ECM quantification assays, Tukey’s or Sidak’s multiple comparison test for the ECM Fn strain (FRET), Tukey’s multiple comparison test for the keratocyte Collagen-1 ECM quantification and the single cell force generation experiments, and Sidak’s multiple comparison test for the HFF Fn and Collagen-1 ECM quantification assays. The level for statistical significance was set at *P* < 0.05 for all comparisons. GraphPad Prism version 6.00 for Windows (GraphPad Software, La Jolla CA, USA, www.graphpad.com) was used for all statistical analyses.

## Supplementary Information

Supplementary Information 1.

Supplementary Information 2.

## Data Availability

The datasets generated and analyzed during the current study are available from the corresponding authors on reasonable request.

## References

[1] Kivanany PB, Grose KC, Tippani M, Su S, Petroll WM (2018). Assessment of corneal stromal remodeling and regeneration after photorefractive keratectomy. Sci. Rep..

[2] Petroll WM, Varner VD, Schmidtke DW (2020). Keratocyte mechanobiology. Exp. Eye Res..

[3] Jester JV (1994). Corneal keratocytes: In situ and in vitro organization of cytoskeletal contractile proteins. Investig. Ophthalmol. Vis. Sci..

[4] Pakshir P (2020). The myofibroblast at a glance. J. Cell Sci..

[5] Jester JV, Ho-Chang J (2003). Modulation of cultured corneal keratocyte phenotype by growth factors/cytokines control in vitro contractility and extracellular matrix contraction. Exp. Eye Res..

[6] Wilson SE, Marino GK, Torricelli AAM, Medeiros CS (2017). Injury and defective regeneration of the epithelial basement membrane in corneal fibrosis: A paradigm for fibrosis in other organs?. Matrix Biol..

[7] Desmouliere A, Redard M, Darby I, Gabbiani G (1995). Apoptosis mediates the decrease in cellularity during the transition between granulation tissue and scar. Am. J. Pathol..

[8] Parker MW (2014). Fibrotic extracellular matrix activates a profibrotic positive feedback loop. J. Clin. Investig..

[9] Walraven M, Hinz B (2018). Therapeutic approaches to control tissue repair and fibrosis: Extracellular matrix as a game changer. Matrix Biol..

[10] Pakshir P, Hinz B (2018). The big five in fibrosis: Macrophages, myofibroblasts, matrix, mechanics, and miscommunication. Matrix Biol..

[11] Franco-Barraza J (2017). Matrix-regulated integrin alpha(v)beta(5) maintains alpha(5)beta(1)-dependent desmoplastic traits prognostic of neoplastic recurrence. Elife.

[12] Van De Water L, Varney S, Tomasek JJ (2013). Mechanoregulation of the myofibroblast in wound contraction, scarring, and fibrosis: Opportunities for new therapeutic intervention. Adv. Wound Care (New Rochelle).

[13] Lickert S (2022). Platelets drive fibronectin fibrillogenesis using integrin alphaIIbbeta3. Sci. Adv..

[14] Suda T, Nishida T, Ohashi Y, Nakagawa S, Manabe R (1981). Fibronectin appears at the site of corneal stromal wound in rabbits. Curr. Eye Res..

[15] Meltendorf C (2007). Corneal femtosecond laser keratotomy results in isolated stromal injury and favorable wound-healing response. Investig. Ophthalmol. Vis. Sci..

[16] Sottile J, Hocking DC (2002). Fibronectin polymerization regulates the composition and stability of extracellular matrix fibrils and cell-matrix adhesions. Mol. Biol. Cell.

[17] Kadler KE, Hill A, Canty-Laird EG (2008). Collagen fibrillogenesis: Fibronectin, integrins, and minor collagens as organizers and nucleators. Curr. Opin. Cell Biol..

[18] Kubow KE (2015). Mechanical forces regulate the interactions of fibronectin and collagen I in extracellular matrix. Nat. Commun..

[19] Vogel V (2018). Unraveling the mechanobiology of extracellular matrix. Annu. Rev. Physiol..

[20] Chabria M, Hertig S, Smith ML, Vogel V (2010). Stretching fibronectin fibres disrupts binding of bacterial adhesins by physically destroying an epitope. Nat. Commun..

[21] Baneyx G, Baugh L, Vogel V (2002). Fibronectin extension and unfolding within cell matrix fibrils controlled by cytoskeletal tension. Proc. Natl. Acad. Sci. U. S. A..

[22] Zhong C (1998). Rho-mediated contractility exposes a cryptic site in fibronectin and induces fibronectin matrix assembly. J. Cell Biol..

[23] Lemmon CA, Chen CS, Romer LH (2009). Cell traction forces direct fibronectin matrix assembly. Biophys. J..

[24] Seong J (2013). Distinct biophysical mechanisms of focal adhesion kinase mechanoactivation by different extracellular matrix proteins. Proc. Natl. Acad. Sci. U. S. A..

[25] Krammer A, Craig D, Thomas WE, Schulten K, Vogel V (2002). A structural model for force regulated integrin binding to fibronectin’s RGD-synergy site. Matrix Biol..

[26] Petroll WM, Miron-Mendoza M (2015). Mechanical interactions and crosstalk between corneal keratocytes and the extracellular matrix. Exp. Eye Res..

[27] Etheredge L, Kane BP, Hassell JR (2009). The effect of growth factor signaling on keratocytes in vitro and its relationship to the phases of stromal wound repair. Investig. Ophthalmol. Vis. Sci..

[28] Kim A, Lakshman N, Karamichos D, Petroll WM (2010). Growth factor regulation of corneal keratocyte differentiation and migration in compressed collagen matrices. Investig. Ophthalmol. Vis. Sci..

[29] Serini G (1998). The fibronectin domain ED-A is crucial for myofibroblastic phenotype induction by transforming growth factor-beta1. J. Cell Biol..

[30] Shiu JY, Aires L, Lin Z, Vogel V (2018). Nanopillar force measurements reveal actin-cap-mediated YAP mechanotransduction. Nat. Cell Biol..

[31] Smith ML (2007). Force-induced unfolding of fibronectin in the extracellular matrix of living cells. PLoS Biol..

[32] Berthaut A (2011). Insulin growth factor promotes human corneal fibroblast network formation in vitro. Investig. Ophthalmol. Vis. Sci..

[33] Jester JV, Brown D, Pappa A, Vasiliou V (2012). Myofibroblast differentiation modulates keratocyte crystallin protein expression, concentration, and cellular light scattering. Investig. Ophthalmol. Vis. Sci..

[34] Balestrini JL, Chaudhry S, Sarrazy V, Koehler A, Hinz B (2012). The mechanical memory of lung myofibroblasts. Integr. Biol. (Camb).

[35] Kollmannsberger P, Bidan CM, Dunlop JWC, Fratzl P, Vogel V (2018). Tensile forces drive a reversible fibroblast-to-myofibroblast transition during tissue growth in engineered clefts. Sci. Adv..

[36] Thomasy SM (2018). Latrunculin B and substratum stiffness regulate corneal fibroblast to myofibroblast transformation. Exp. Eye Res..

[37] Hinz B (2007). The myofibroblast: One function, multiple origins. Am. J. Pathol..

[38] Dubin-Thaler BJ (2008). Quantification of cell edge velocities and traction forces reveals distinct motility modules during cell spreading. PLoS ONE.

[39] Kubow KE (2009). Crosslinking of cell-derived 3D scaffolds up-regulates the stretching and unfolding of new extracellular matrix assembled by reseeded cells. Integr. Biol. (Camb).

[40] Ritter SJ, Davies PJ (1998). Identification of a transforming growth factor-beta1/bone morphogenetic protein 4 (TGF-beta1/BMP4) response element within the mouse tissue transglutaminase gene promoter. J. Biol. Chem..

[41] Nurminskaya MV, Belkin AM (2012). Cellular functions of tissue transglutaminase. Int. Rev. Cell Mol. Biol..

[42] Buhren J (2009). Optical effects of anti-TGFbeta treatment after photorefractive keratectomy in a cat model. Investig. Ophthalmol. Vis. Sci..

[43] Moller-Pedersen T, Li HF, Petroll WM, Cavanagh HD, Jester JV (1998). Confocal microscopic characterization of wound repair after photorefractive keratectomy. Investig. Ophthalmol. Vis. Sci..

[44] Wilson SE (2020). Corneal wound healing. Exp. Eye Res..

[45] Kivanany PB (2018). An in vitro model for assessing corneal keratocyte spreading and migration on aligned fibrillar collagen. J. Funct. Biomater..

[46] Miron-Mendoza M, Graham E, Manohar S, Petroll WM (2017). Fibroblast-fibronectin patterning and network formation in 3D fibrin matrices. Matrix Biol..

[47] Pankov R (2005). A Rac switch regulates random versus directionally persistent cell migration. J. Cell Biol..

[48] Bhattacharyya S, Midwood KS, Yin H, Varga J (2017). Toll-like receptor-4 signaling drives persistent fibroblast activation and prevents fibrosis resolution in scleroderma. Adv. Wound Care (New Rochelle).

[49] Nia HT, Munn LL, Jain RK (2020). Physical traits of cancer. Science.

[50] Saini K, Cho S, Dooling LJ, Discher DE (2020). Tension in fibrils suppresses their enzymatic degradation – A molecular mechanism for ‘use it or lose it’. Matrix Biol..

[51] Benn MC (2023). How the mechanobiology orchestrates the iterative and reciprocal ECM-cell cross-talk that drives microtissue growth. Sci. Adv..

[52] Fu J (2010). Mechanical regulation of cell function with geometrically modulated elastomeric substrates. Nat. Methods.

[53] Lakshman N, Petroll WM (2012). Growth factor regulation of corneal keratocyte mechanical phenotypes in 3-D collagen matrices. Investig. Ophthalmol. Vis. Sci..

[54] Myrna KE (2012). Substratum topography modulates corneal fibroblast to myofibroblast transformation. Investig. Ophthalmol. Vis. Sci..

[55] Maruri DP (2020). ECM stiffness controls the activation and contractility of corneal keratocytes in response to TGF-beta1. Biophys. J..

[56] Chaudhuri O, Cooper-White J, Janmey PA, Mooney DJ, Shenoy VB (2020). Effects of extracellular matrix viscoelasticity on cellular behaviour. Nature.

[57] Yang S, Zhang J, Tan Y, Wang Y (2022). Unraveling the mechanobiology of cornea: From bench side to the clinic. Front. Bioeng. Biotechnol..

[58] Raghunathan VK (2017). Tissue and cellular biomechanics during corneal wound injury and repair. Acta Biomater..

[59] Jester JV, Barry-Lane PA, Cavanagh HD, Petroll WM (1996). Induction of alpha-smooth muscle actin expression and myofibroblast transformation in cultured corneal keratocytes. Cornea.

[60] Zhang Y (2014). Disentangling the multifactorial contributions of fibronectin, collagen and cyclic strain on MMP expression and extracellular matrix remodeling by fibroblasts. Matrix Biol..

[61] Benn, M. C. FIJI-macro_zStackQuant. https://github.com/BennSynergy/FIJI-macro_zStackQuant.git, 10.5281/zenodo.7978897.

[62] Fruh SM, Schoen I, Ries J, Vogel V (2015). Molecular architecture of native fibronectin fibrils. Nat. Commun..

[63] Kadler KE (2017). Fell muir lecture: Collagen fibril formation in vitro and in vivo. Int. J. Exp. Pathol..

[64] Antia M, Baneyx G, Kubow KE, Vogel V (2008). Fibronectin in aging extracellular matrix fibrils is progressively unfolded by cells and elicits an enhanced rigidity response. Faraday Discuss..

