# Supplementary material for: Growth factors and mechano-regulated reciprocal crosstalk with extracellular matrix tune the keratocyte–fibroblast/myofibroblast transition
Source: Sci Rep. 2023 Jul 13;13:11350. doi: 10.1038/s41598-023-37776-9 (PMC10345140; doi:10.1038/s41598-023-37776-9)
Supplement: Supplementary file 1 — Supplementary Information 1. [file 41598_2023_37776_MOESM1_ESM.docx]

Supplementary Information

Growth Factors and Mechano-Regulated Reciprocal Crosstalk with Extracellular Matrix tune the Keratocyte-Fibroblast/Myofibroblast Transition

Simon A. Pot^1, 4#^*, Zhe Lin^2#^, Jauye Shiu^1, 3^, Mario C. Benn^1^, Viola Vogel^1^*

^1^ Laboratory of Applied Mechanobiology, Department of Health Sciences and Technology,
ETH Zurich, Vladimir-Prelog-Weg 4, 8093 Zurich, Switzerland. Email: mario.benn@hest.ethz.ch, viola.vogel@hest.ethz.ch

^2^ Ruisi (Fujian) Biomedical Engineering Research Center Co Ltd, 26-1 Wulongjiang Road, Fuzhou, 350100 P. R. China. Email: admin@linzhe.com

^3^ Graduate Institute of Biomedical Sciences, China Medical University, No. 91, Xueshi Rd, North District, Taichung City, Taiwan. Email: jyshiu@mail.cmu.edu.tw

^4^ Ophthalmology Section, Vetsuisse Faculty, University of Zurich, Winterthurerstrasse 260, 8057 Zurich, Switzerland. Email: spot@vetclinics.uzh.ch

^#^ SAP and ZL have contributed equally to this work

* SAP and VV share corresponding authorship for this publication.

Simon A. Pot: spot@vetclinics.uzh.ch

Viola Vogel: viola.vogel@hest.ethz.ch

Supplementary figures and table


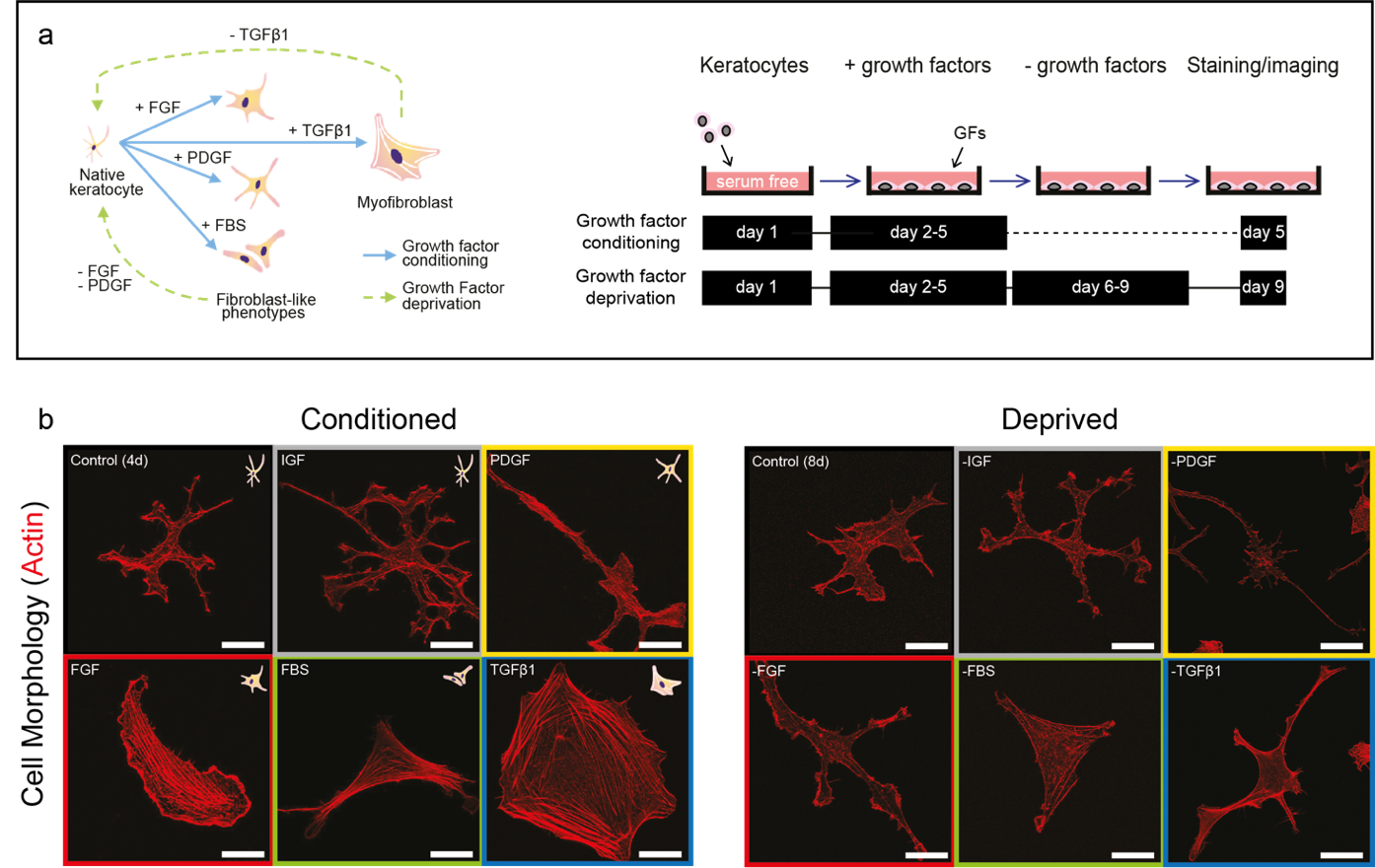


**Figure S1:** **Keratocyte to fibroblast/myofibroblast (K-F/M) phenotype transition and reversal in growth factor-conditioned and subsequently deprived primary corneal keratocytes.**

In panel (a) a brief overview of the keratocyte-fibroblast/myofibroblast (K-F/M) phenotype transition process *in vitro* is provided and the experimental timeline is visualized. One day of cell attachment in serum-free culture medium on Fn-coated planar glass tissue culture substrates was followed by four days of growth factor exposure to various growth factors (insulin-like growth factor (IGF-1), platelet-derived growth factor (PDGF), fibroblast growth factor (FGF), transforming growth factor β1 (TGFβ1)) or fetal bovine serum (FBS), then fixation, staining and imaging. In the growth factor deprivation experiments the four days of growth factor exposure were followed by four days of growth factor deprivation. In the lower panels (b) the effect of exposure to various growth factors on actin cytoskeletal morphology is demonstrated. Control and IGF-1: dendritic shape, minimal and exclusively cortical expression of actin. PDGF: elongated shape, cortical actin expression. FGF or FBS: typical fibroblast-like morphology, spindle shaped cell body, cortical actin expression and actin stress fiber assembly. TGFβ1: typical myofibroblast morphology, large cell body, cortical actin expression and extensive actin stress fiber assembly. A four-day period of growth factor deprivation after growth factor conditioning resulted in a reversion towards the dendritic control keratocyte morphology and a disassembly of cytoskeletal stress fibers, apart from cortical fibers lining the cell edges in PDGF and FGF conditioned keratocytes. Some of the PDGF-deprived cells retained their elongated features. Most FBS and TGFβ1 conditioned keratocytes still expressed some basal stress fibers after four days of FBS/TGFβ1 deprivation. Red = actin. Scale bars: 25µm. The various phenotypes have been color coded in the relevant figures and graphs throughout the manuscript: native keratocyte control = black, IGF-1 = grey, PDGF = orange, FGF = red, FBS = green, TGFβ1 = blue.


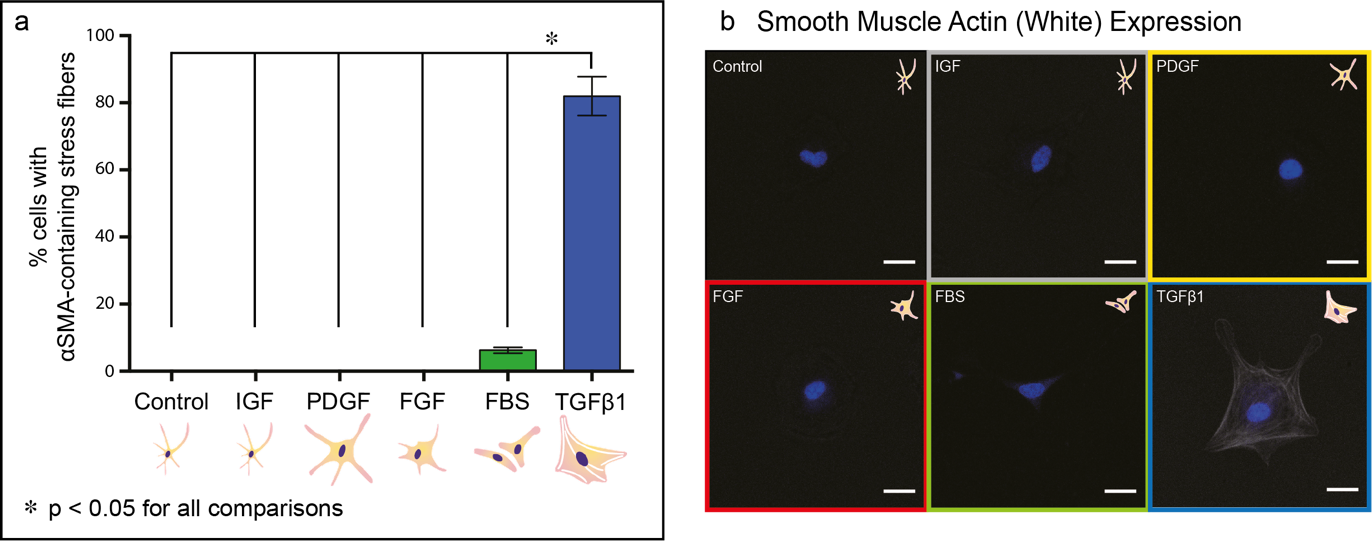


**Figure S2: Quantification of αSMA containing stress fibers in growth factor conditioned keratocytes.**

(a) Assembly of αSMA into stress fibers was observed in 6.2% of FBS conditioned and 82% of TGFβ1 conditioned keratocytes. αSMA containing stress fibers were not observed in growth factor deprived keratocytes. (b) Growth factor conditioned keratocytes: αSMA immunocytochemistry. White = αSMA, Blue = DAPI. Scale bars: 25µm. Statistical comparisons were performed via one-way ANOVA and Holm-Sidak’s multiple comparison tests with significance set at p < 0.05 for all comparisons.

**Table S1: rtPCR primers**

| Gene | Accession # | Primer | Primer | Expected size (bp) |
| --- | --- | --- | --- | --- |
| Keratocan | DQ239829.1 | GTCTCACAATCGCCTCACAA | GGTCCATGGATGAACGAATC | 153 |
| ALDH1a1 | AY038801.1 | ACTCCCCTCACTGCTCTTCA | AACACTGGCCCTGATGGTAG | 316 |
| αSMA | X60732.1 | TGCTGTCCCTCTATGCCTCT | GAAGGAATAGCCACGCTCAG | 148 |
| Β-actin | AF309819.1 | ATCGTGATGGACTCCGGCGAC | AGCGCCACGTAGCACAGC | 211 |


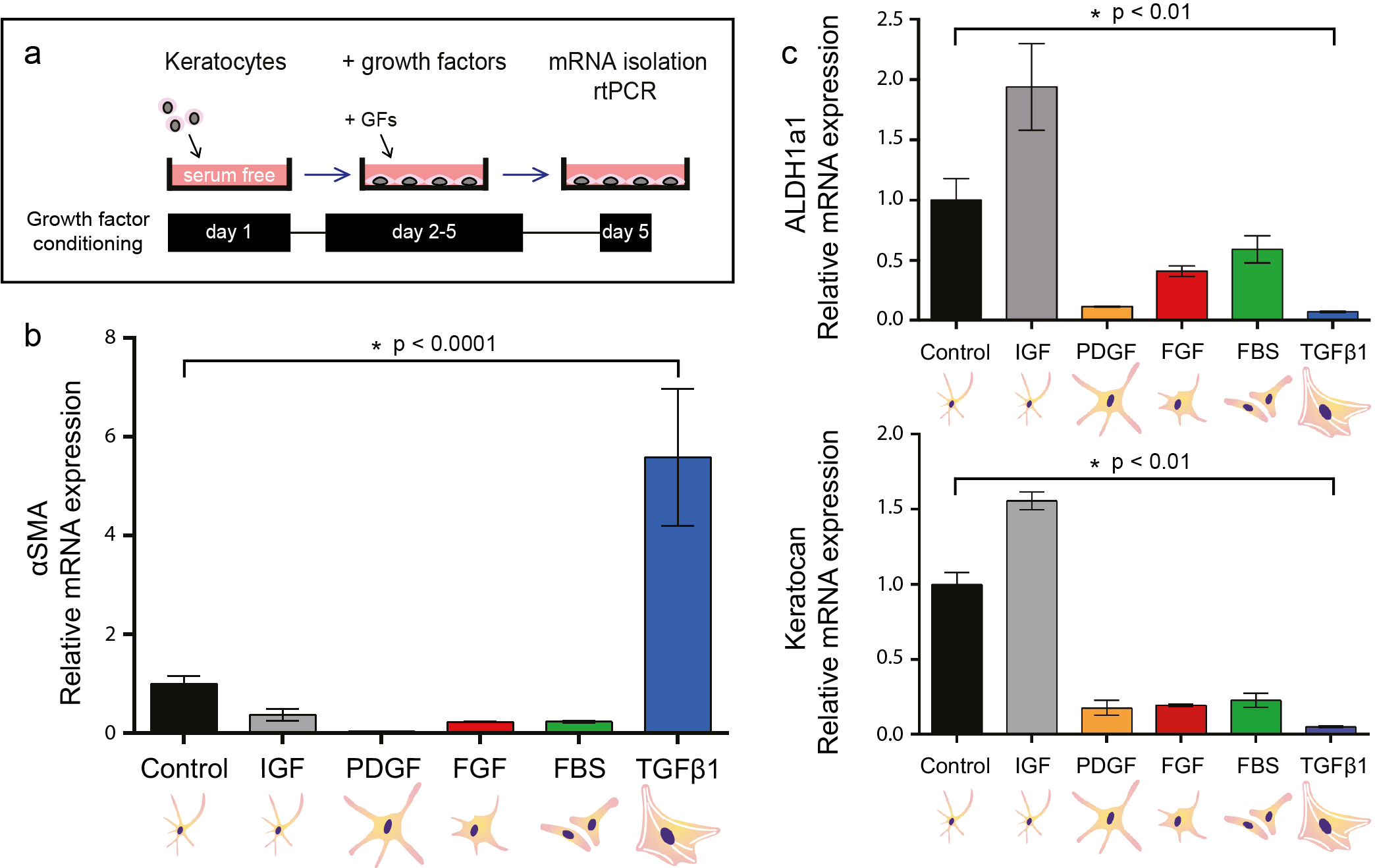


**Figure S3: Real time PCR quantification of αSMA, keratocan and ALDH1a1 mRNA expression in growth factor conditioned keratocytes.**

Real-time PCR was used to quantify the expression of αSMA, keratocan and ALDH1a1 mRNA. Keratocytes play an important role in the maintenance of corneal transparency through active expression of water-soluble cytoplasmic proteins, called crystallins, and ECM components, like keratocan. The particularly high cytoplasmic concentration of crystallin proteins (e.g. aldehyde dehydrogenase-1a1) in keratocytes elevates the refractive index (RI) of their cytoplasm. This effectively equalizes the RIs of the keratocyte cytoplasm and surrounding ECM, thereby minimizing light scatter at the cell-ECM interface^1^. As such, ALDH1a1 and keratocan are keratocyte markers. (a) Experimental timeline: one day of cell attachment in serum free culture medium, four days of keratocyte exposure to various growth factors (IGF-1, PDGF, FGF, FBS or TGFβ1), then mRNA isolation and sample processing for rtPCR. (b) Alpha-SMA expression was significantly increased in TGFβ1 conditioned myofibroblasts (p < 0.0001) compared to control keratocytes. The decreased αSMA expression in the other keratocyte phenotypes was not statistically significant compared to control keratocytes. (c) The expression of keratocan and ALDH1a1 was significantly decreased in TGFβ1 conditioned myofibroblasts (p < 0.01) compared to control keratocytes. Differences in keratocan and ALDH1a1 expression in the other keratocyte phenotypes were not statistically significant compared to control keratocytes. The experiment was performed in triplicate with duplicate samples, with the results of one representative experiment shown here. The data from all three experiments were pooled for statistical comparison.


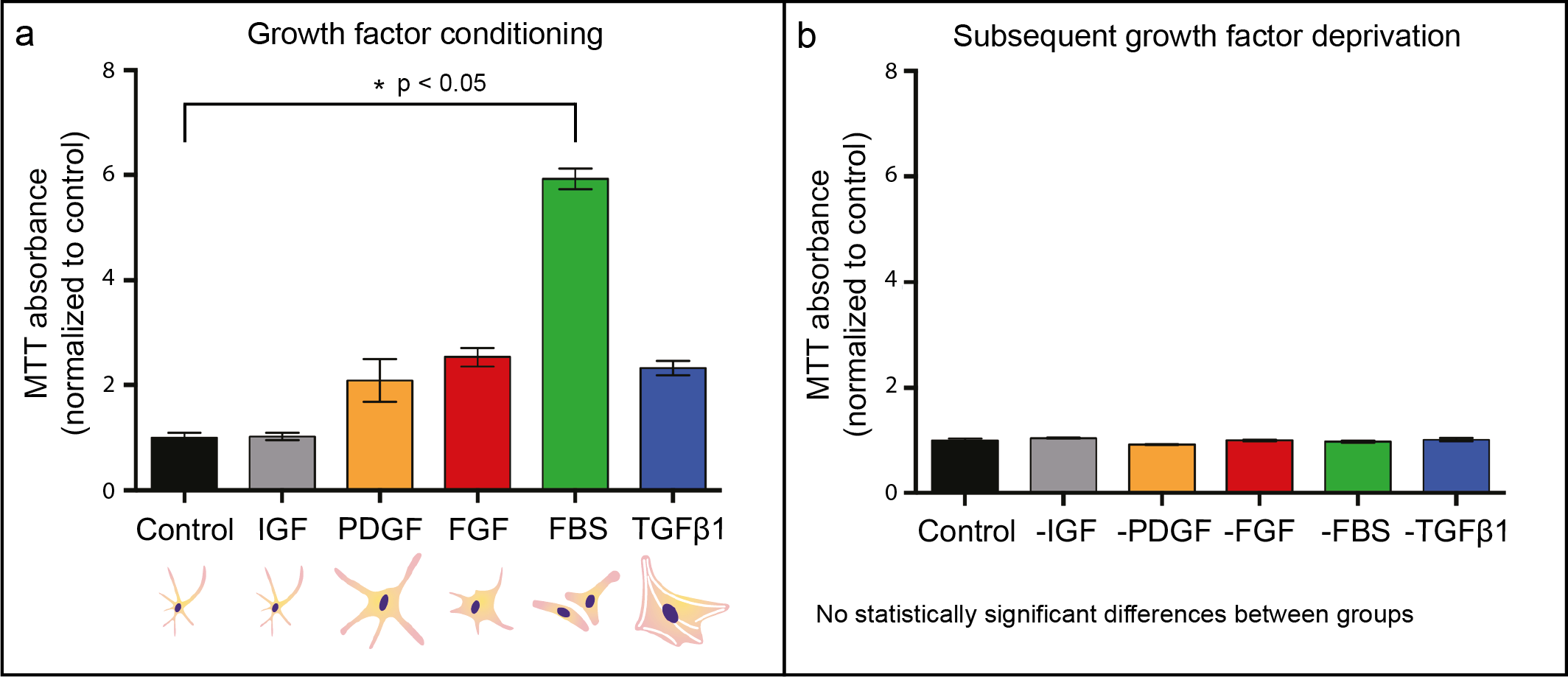


**Figure S4: Keratocyte metabolic activity increased following growth factor conditioning, and decreased again following growth factor deprivation.**

Cell metabolic activity was measured via MTT assay as an indicator of cell proliferation in growth factor conditioned keratocytes between culture days 2 and 5, and in growth factor deprived keratocytes between culture days 6 and 9. (a) PDGF, FGF, FBS and TGFβ1 conditioned keratocytes demonstrated an increase in metabolic activity compared to control keratocytes (p<0.05 for FBS conditioned compared to control keratocytes). (b) A reversion of cell metabolic activity to the level of native keratocytes was observed in all phenotypes after a four-day period of growth factor deprivation. Statistical comparisons were performed via Kruskal–Wallis one-way ANOVA for nonparametric data and Dunn’s multiple comparisons tests with significance set at p < 0.05 for all comparisons.


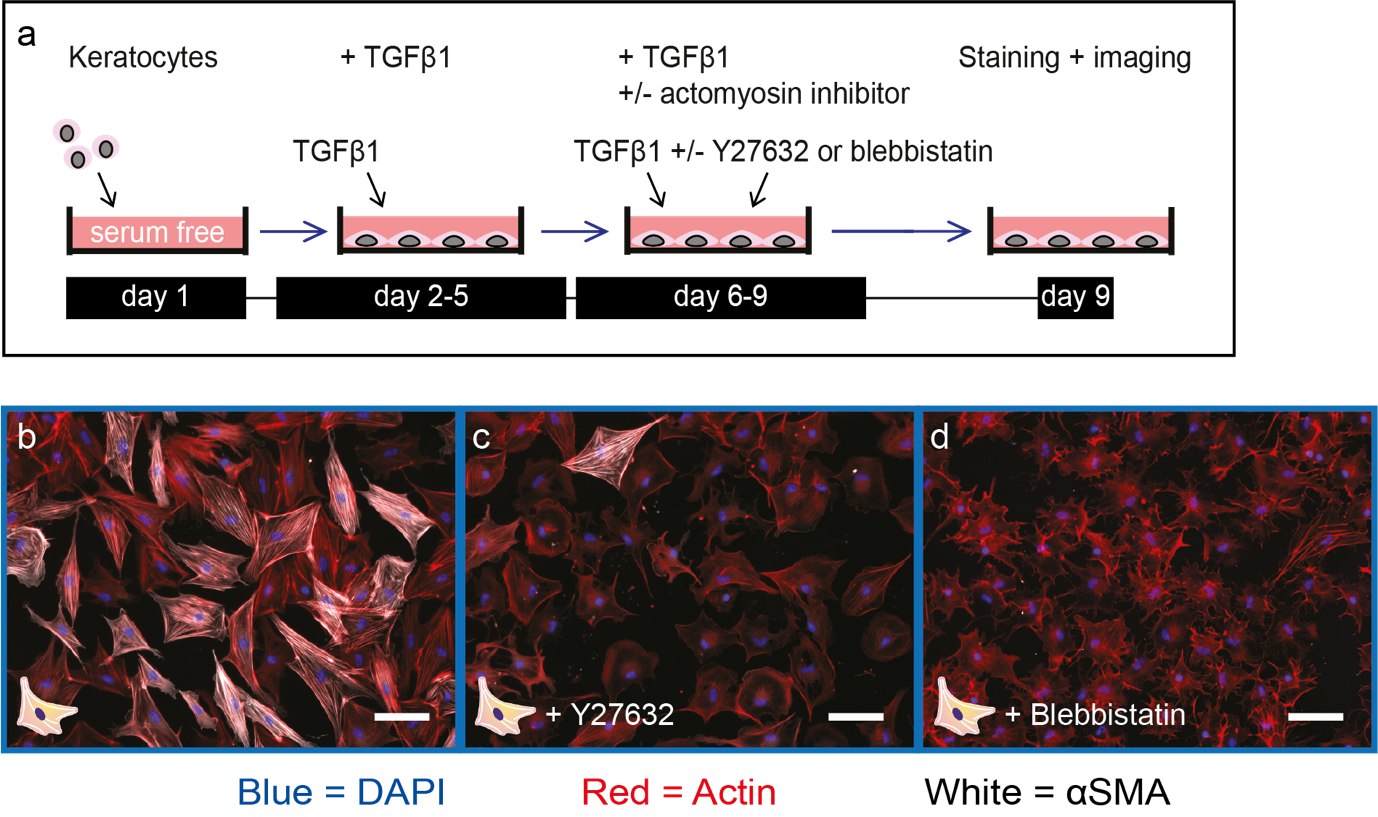


**Figure S5**: **A functional contractile actomyosin cytoskeleton is necessary to support expression of αSMA containing stress fibers.**

(a) Experimental timeline: one day of cell attachment in serum free culture medium, four days of keratocyte exposure to TGFβ1, followed by four days of exposure to a ROCK inhibitor (Y-27632) or a myosin-II inhibitor (blebbistatin) in the continued presence of TGFβ1 in the culture medium, fixation and sample processing for imaging. (b) The typical morphology of myofibroblasts cultured on a rigid planar substrate: large cell body, thick actin stress fibers spanning the entire cell body, αSMA assembly into a large proportion of the actin stress fibers. (c) The addition of ROCK inhibitor Y-27632 to the culture medium of TGFβ1 conditioned keratocytes caused an almost complete disappearance of αSMA from the cytoskeleton and a strong decrease in the number of stress fibers. Most stress fibers that remained present were peripheral fibers concentrated in the cortical region, few cells with thin fibers spanning the cell body remained. The general spread-out morphology of the cell body was maintained. (d) The addition of myosin-II inhibitor blebbistatin to the culture medium of TGFβ1 conditioned keratocytes caused a complete disappearance of αSMA from the cytoskeleton and an almost complete disappearance of stress fibers. Disruption of circumferential actin bundles and a reversion to a stellate cellular morphology were observed. Blue = DAPI, red = F-actin, white = αSMA. Scale bars: 100μm.


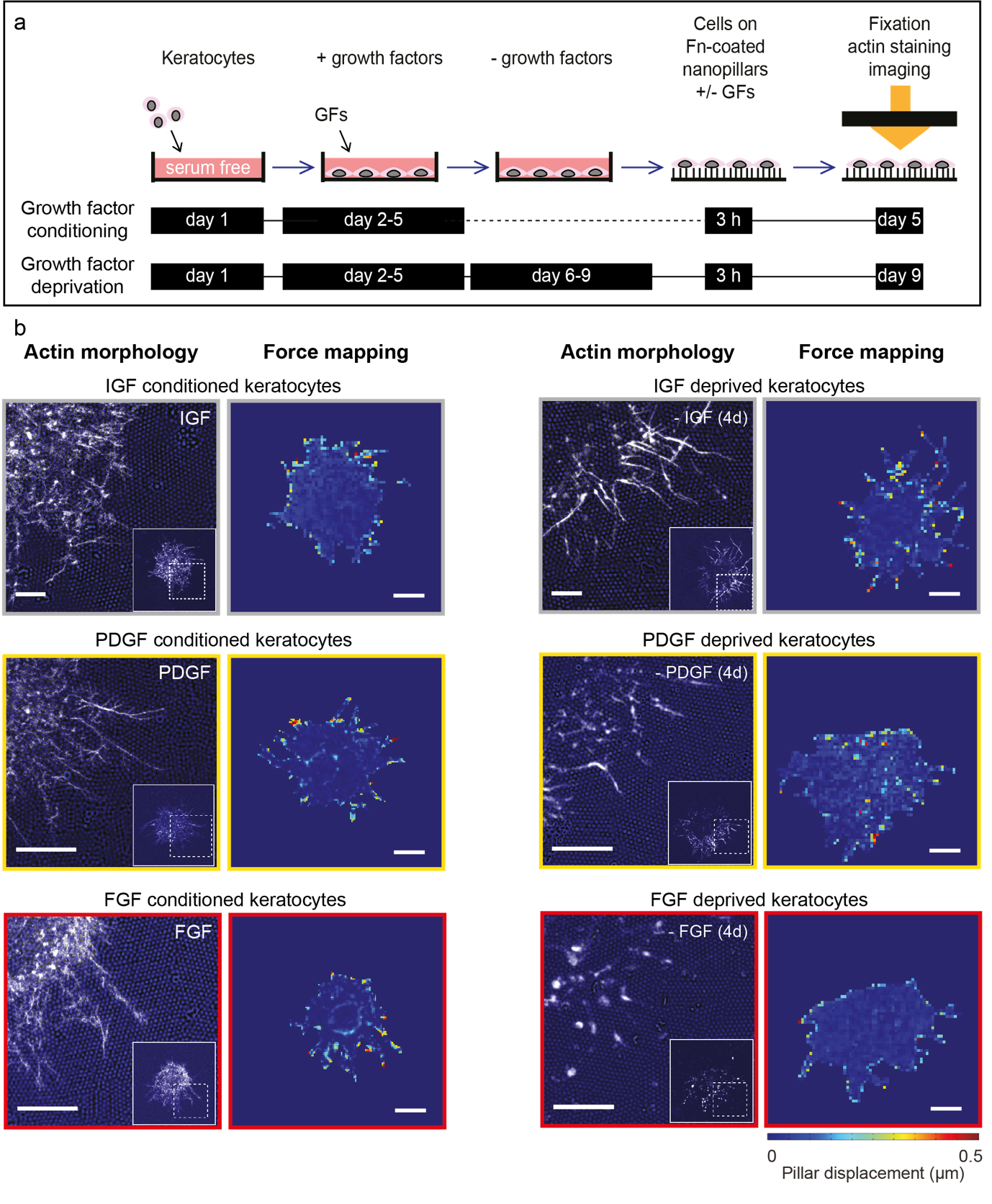
**Figure S6: Actin cytoskeleton morphology and force distribution of IGF-1, PDGF and FGF conditioned, then deprived keratocytes seeded on nanopillar substrates.**

(a) Experimental timeline: growth factor conditioned and growth factor conditioned and subsequently deprived keratocytes were seeded onto Fn-coated nanopillar substrates, fixated three hours after seeding, then phalloidin stained and imaged. Pillar displacement was analyzed with particle tracking software on confocal microscopic images. (b) Actin cytoskeleton morphology of IGF-1, PDGF and FGF conditioned and conditioned and subsequently deprived keratocytes seeded on nanopillar substrates. Scale bars: 5 μm. Colorimetric distribution maps of force induced pillar displacement across the cell body of corresponding cells are juxtapositioned. Scale bars: 20 μm. The displacement of single nanopillars was topographically mapped with the level of displacement indicated by colors ranging from dark blue (0 μm displacement) to dark red (0.5 μm displacement). The morphology of IGF-1, PDGF and FGF conditioned and conditioned and subsequently deprived keratocytes on nanopillar substrates was identical to the morphology of native keratocytes (Figure 2b).


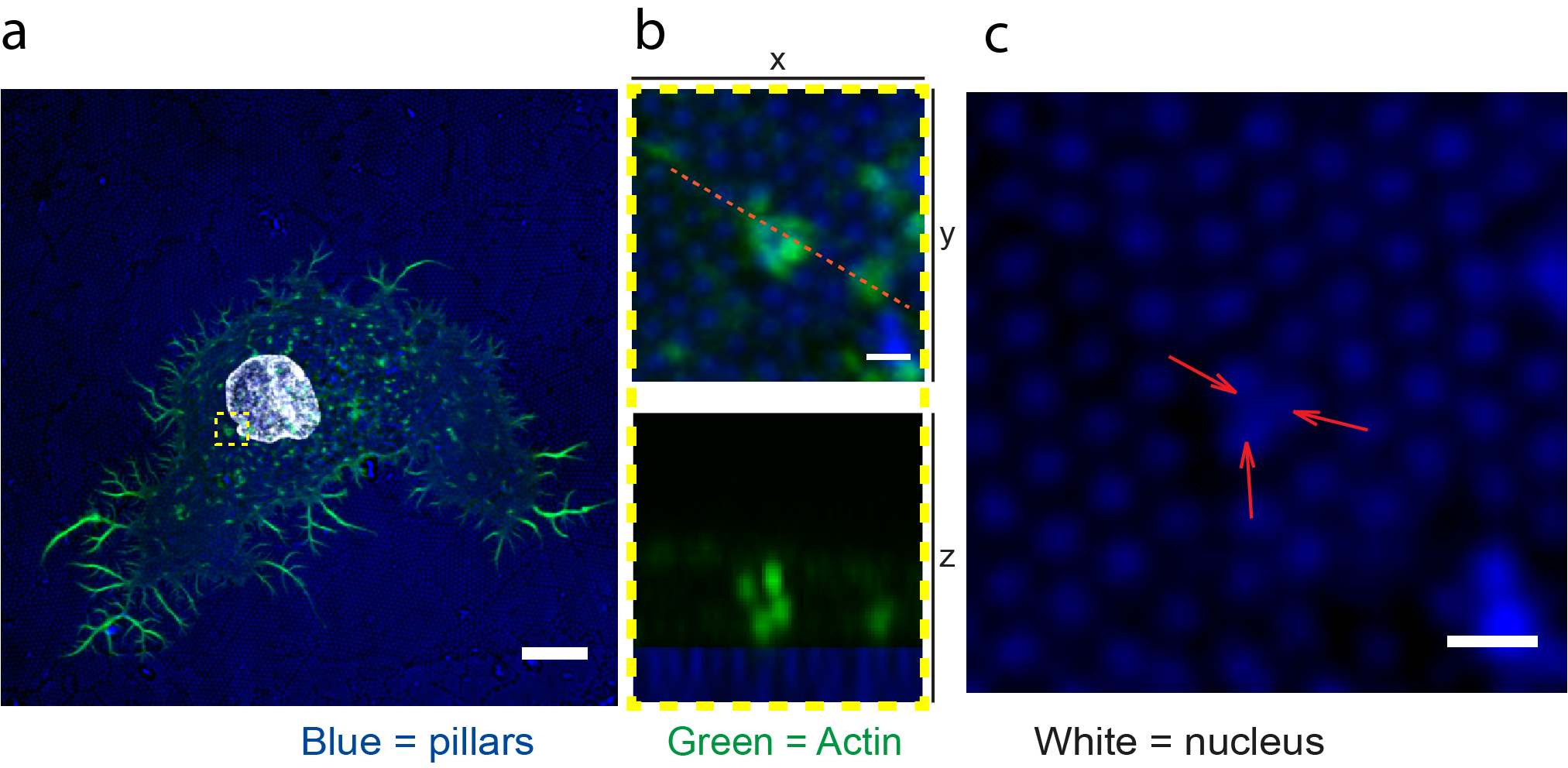


**Figure S7: TGFβ1 conditioned keratocytes cultured on nanopillar substrates express nodular actin structures colocalized with hotspots of force generation.**

TGFβ1 conditioned keratocytes were fixed and fluorescently labeled with actin (green) and DAPI (white) after a 3-hour culture period on Fn-coated nanopillar substrates (fluorescently labeled: blue). (a) TGFβ1 conditioned keratocytes on nanopillar substrates formed narrow, dendritic filopodial protrusions at the cell edges, and vertical, nodular, doughnut-shaped actin structures throughout the central cell body. Scale bar: 10 µm. (b) Zoom-in (x-y) from the boxed-in yellow region in (a); the z-projection represents a cross-sectional view along the dashed red line in the x-y image. Scale bar: 1 µm. (c) Zoom-in from x-y in (b) illustrating nanopillars combined with force vectors, highlighting colocalization of the observed actin structures with subtending areas of increased pillar deflection. A cluster of three to four nanopilllars were typically pulled together directly underneath the nodular actin structures. Scale bar: 1 µm.

**
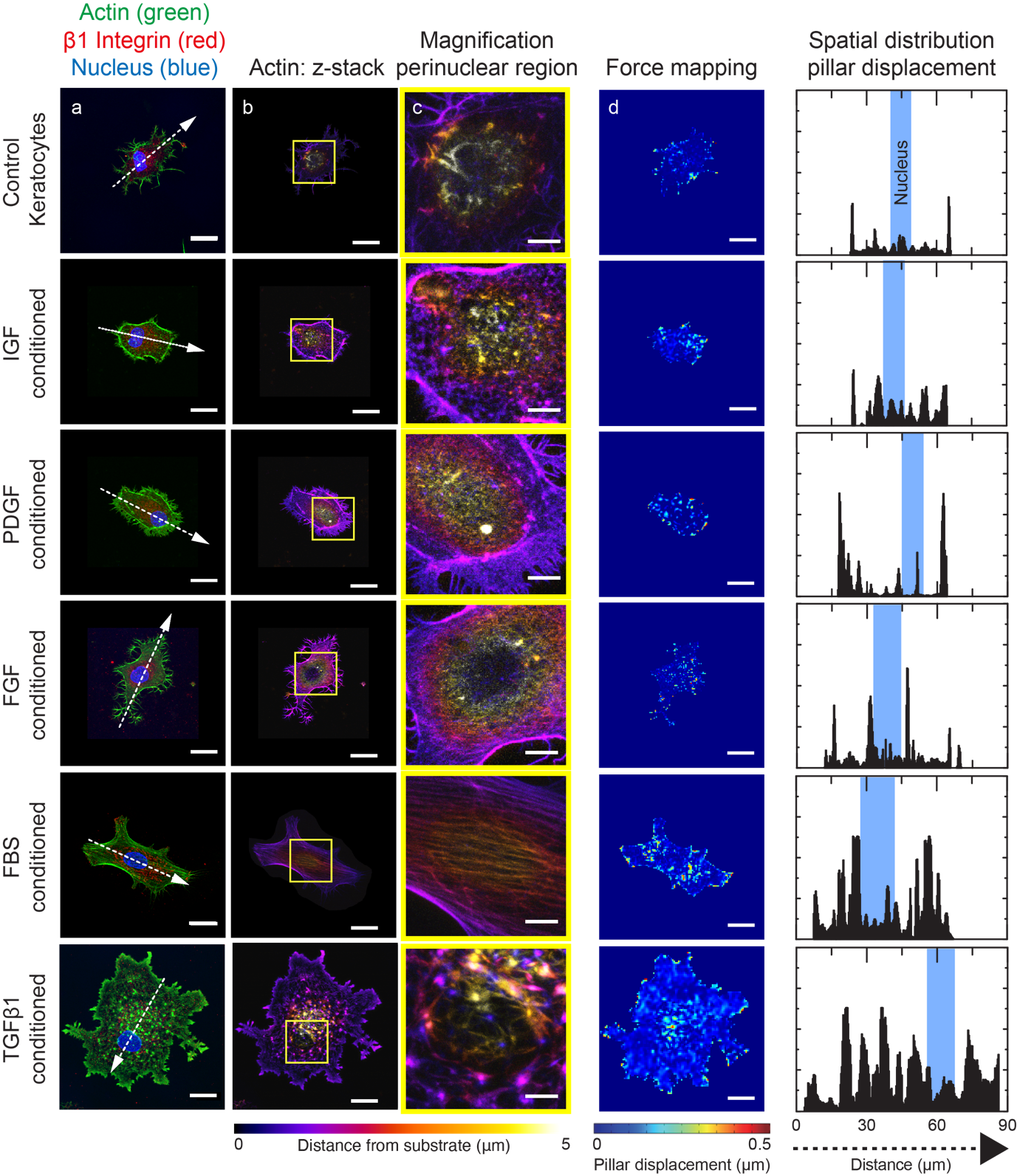
**

**Figure S8: TGFβ1 and FBS conditioning cause a reorganization of the actin cytoskeleton and a redistribution of cell to surface attachments in keratocytes cultured on nanopillar topography.**

Growth factor conditioned keratocytes were fixed and fluorescently labeled with actin (green), β1 integrin (red) and DAPI (blue) after a 3-hour culture period on Fn-coated nanopillar substrates. (a-c) The first three columns of panels illustrate the actin cytoskeleton morphology. (c) The actin morphology in the perinuclear region was presented as boxed-in yellow zoom-in (scale bars column c: 5 μm) from the boxed-in yellow regions in the actin z-stack images in column b. The distance from the substrate was indicated by colors ranging from black (0 μm) to white (5 μm). (d) Corresponding colorimetric maps of the distribution across the cell body of forces exerted on single nanopillars (see Fig. 2) are shown in the fourth column of panels. The displacement of single nanopillars was topographically mapped with the level of displacement indicated by colors ranging from dark blue (0 μm displacement) to dark red (0.5 μm displacement). Scale bars columns a, b, d: 20 μm. For direct comparison, the far right column of panels represents aligned pillar displacement histograms from the regions of interest outlined by the white arrows in the confocal images in column a. The blue rectangular overlays represent the estimated position of the nucleus.

**
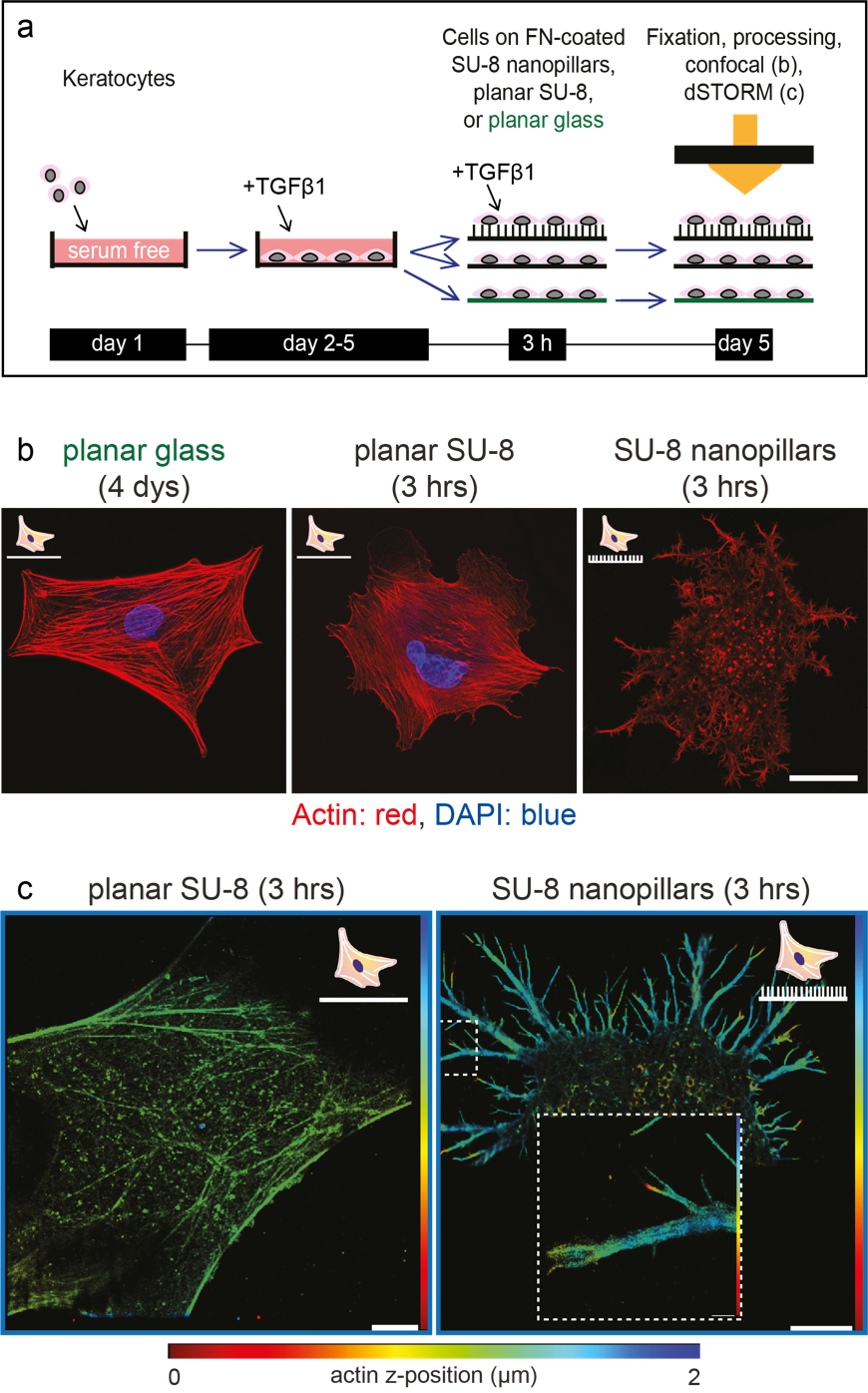
Figure S9: Actin stress fibers are expressed by TGFβ1 conditioned keratocytes cultured on rigid planar, but not on nanopillar substrates.**

(a) Experimental timeline: TGFβ1 conditioned keratocytes were seeded onto Fn-coated SU-8 nanopillar substrates, or onto Fn-coated planar SU-8 or glass substrates. Cells were maintained on glass substrates for four days. The cells were fixated three hours after seeding onto the SU-8 substrates, then phalloidin stained and imaged via confocal or direct stochastic optical reconstruction microscopy (dSTORM). (b, c) TGFβ1 conditioned keratocytes cultured on planar glass and SU-8 substrates expressed the typical actin stress fiber-rich myofibroblast morphology, with broad lamellipodial cell extensions containing a fine actin fiber network visible on dSTORM. In comparison, TGFβ1 conditioned keratocytes cultured on SU-8 nanopillar substrates formed narrow, dendritic filopodial protrusions. b - scale bar: 25 µm; c - scale bars: 2.5 µm, the distance from the substrate was indicated by colors ranging from dark red (0 μm) to dark blue (2 μm) in the dSTORM images.


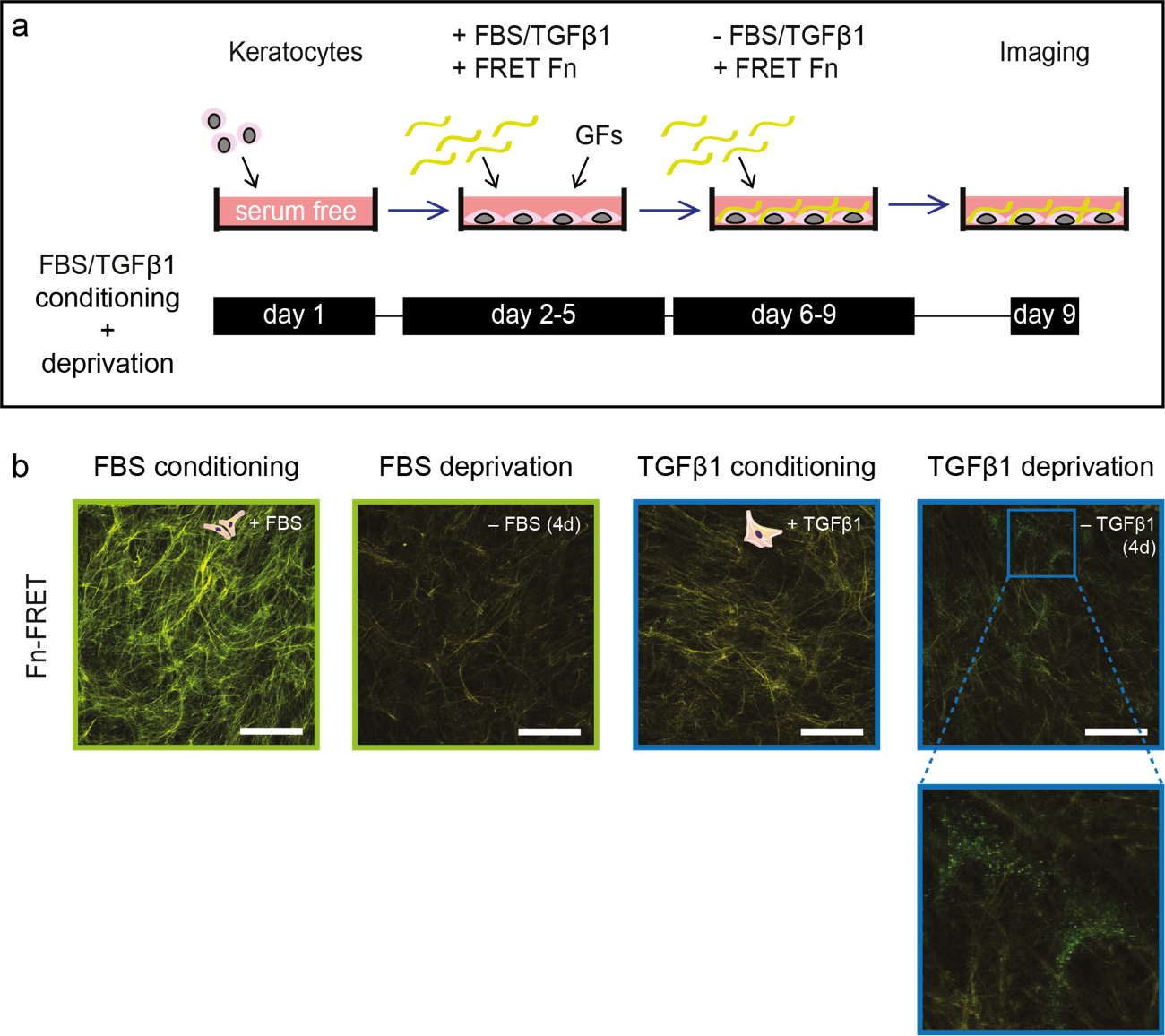


**Figure S10: FBS and TGFβ1 deprivation reduce fibronectin ECM density.**

(a) Experimental timeline: as described in figure 4, but with the addition of a four-day period of FBS/TGFβ1 deprivation following FBS/TGFβ1 conditioning. FRET Fn is added with each culture medium change during the FBS/TGFβ1 conditioning and deprivation periods. (b) Images of FRET Fn ECM for FBS deprived (green) and TGFβ1 deprived (blue) keratocytes demonstrate a considerably less dense Fn ECM compared to FBS and TGFβ1 conditioned keratocytes. Scale bars: 50 μm. Fine globular aggregations of Fn exhibiting a lower Fn-FRET ratio (I_A_/I_D_) compared to the surrounding fibrillar matrix Fn (green vs. yellow fluorescence of surrounding Fn fibers) were observed in all TGFβ1-deprived samples (magnified in lower right image).


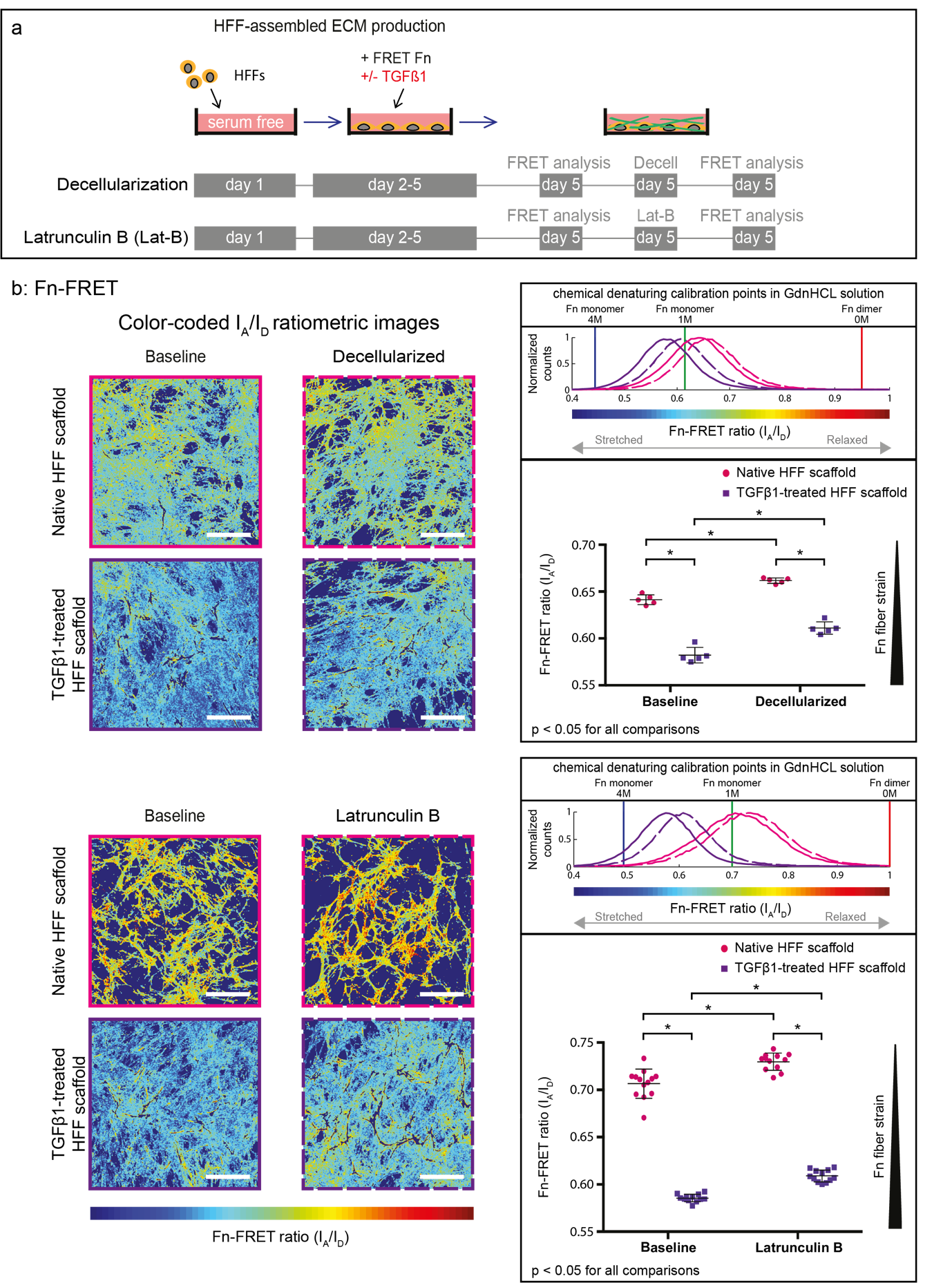


**Figure S11: Residual ECM strain is observed after abolishment of cell-generated forces.**

(a) Experimental timeline: one day of cell attachment followed by four days of culture during which human foreskin fibroblasts (HFFs) were exposed to standard (native HFFs) or TGFβ1-enriched culture medium (TGFβ1-treated HFFs). FRET Fn was added to the culture medium in both groups. On day 5 the HFF-derived ECM samples were decellularized or treated with latrunculin-B to abolish cell-generated forces by removing the cells from the scaffolds or disrupting the actin cytoskeleton, respectively. FRET analyses were performed before (‘baseline’) and after decellularization/latrunculin-B treatment (‘decellularized’ or ‘latrunculin B’). (b) A Fn rich ECM was assembled in all groups. FRET analyses demonstrated a large difference in Fn strain between the ECM derived from native HFFs and from TGFβ1-treated HFFs. A relatively modest release of Fn strain was observed after decellularization and after latrunculin-B treatment in the native HFF and in the TGFβ1-treated HFF assembled ECM. A large residual difference in matrix Fn strain was observed between the native HFF and TGFβ1-treated HFF assembled ECM. From this we conclude that the abolishment of cell generated forces contributed to a release of Fn fiber strain, consistent with previously published data^2^. However, a significant residual matrix strain was preserved in the TGFβ1-treated HFF and possibly in the native HFF ECM. These histograms were derived from one representative field of view from one of three separate experiments in each group. Solution denaturation values for dimeric Fn-DA in 0M GdnHCl (Decell: 0.95, Lat-B: 1) and monomeric Fn-DA in 1M (Decell: 0.62, Lat-B: 0.7) and 4M GdnHCl (Decell: 0.44, Lat-B: 0.49) are shown as vertical red, green, and blue lines, respectively. The scatter plots show mean FRET intensity ratios and were constructed from data averaged from five random fields of view each from three separate experiments per group. The bars signify the means and the whiskers the standard deviation from the mean. Statistical comparisons were performed via one-way ANOVA and Sidak’s multiple comparisons tests with significance set at p < 0.05 for all comparisons. Scale bars: 50 μm.


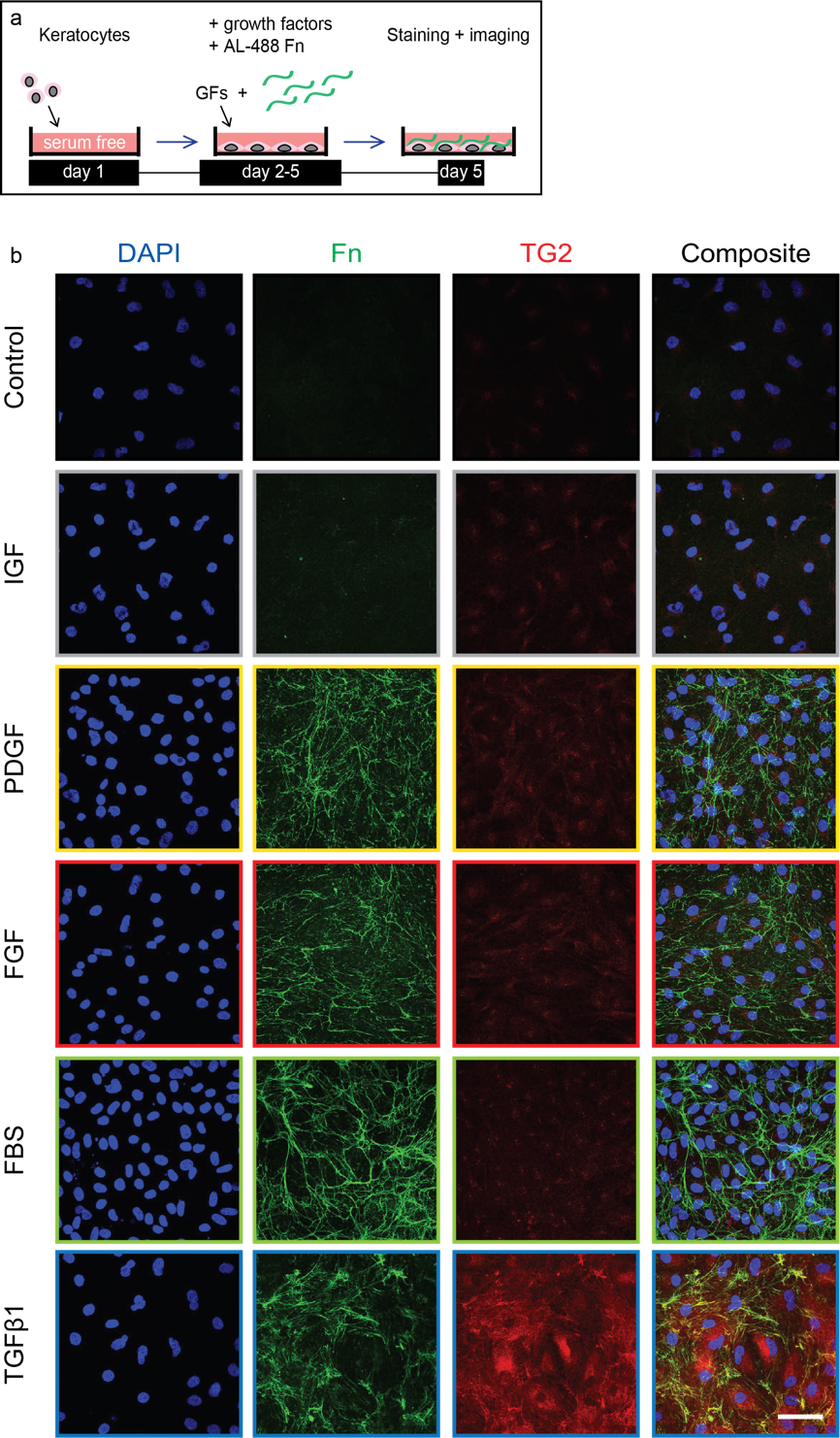
**Figure S12: Transglutaminase 2 (TG2) expression by growth factor conditioned keratocytes.**

(a): Experimental timeline: one day of cell attachment, four days of growth factor conditioning with Alexa-488 fluorescently labeled plasma fibronectin added to the culture medium, then fixation and sample processing for imaging. (b) Fn fibrillogenesis was observed following PDGF, FGF, FBS and TGFβ1 conditioning. Strong positive staining for TG2 was only observed in TGFβ1 conditioned cells, whereas cells exposed to the other growth factors showed minimal TG2 staining. This demonstrates that TGFβ1 conditioning upregulates TG2 in activated keratocytes, as was previously described^3^. blue = DAPI, green = Fn, red = TG2. Scale bar: 50 μm.


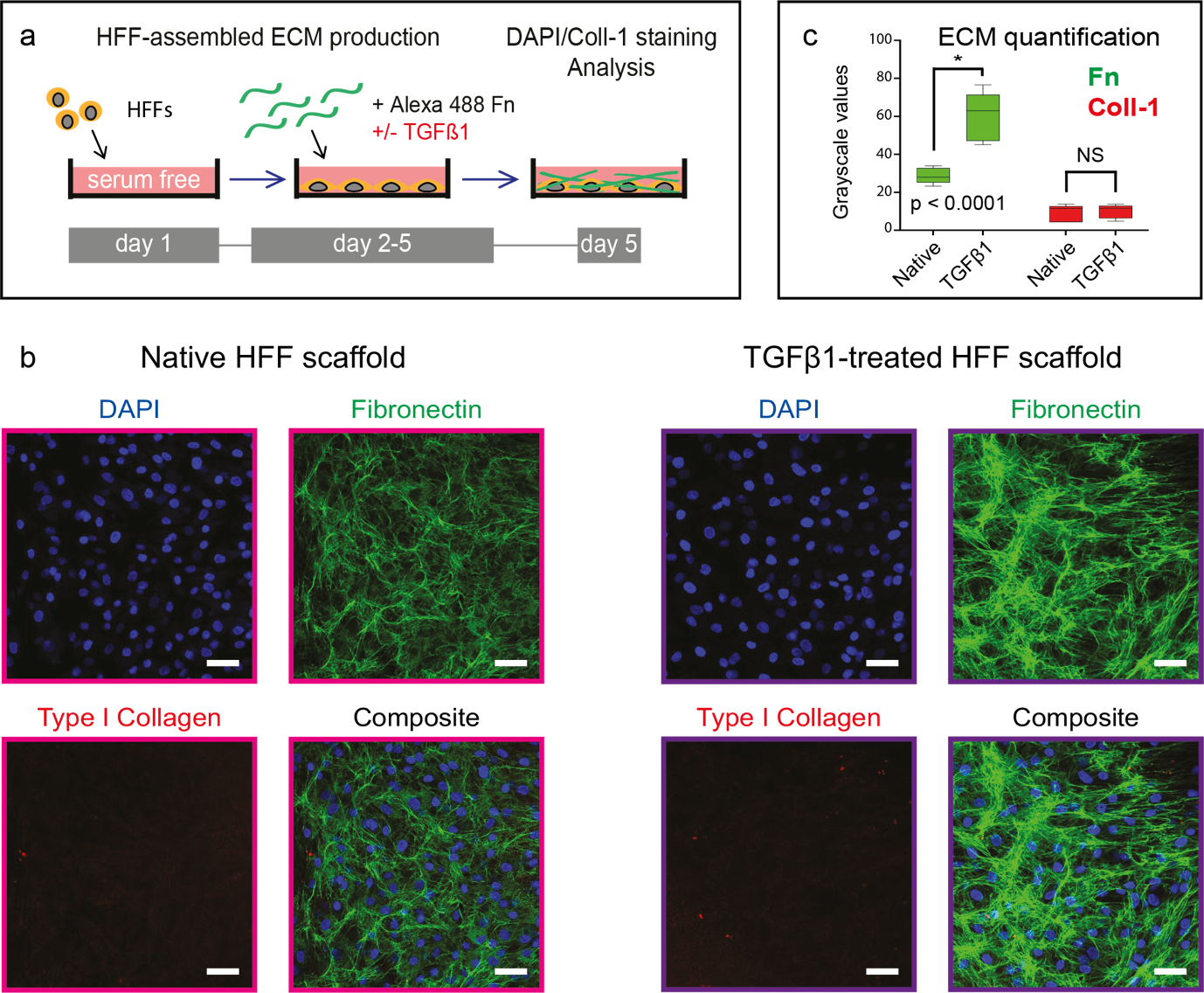


**Figure S13: Fn and collagen-1 content of ECM scaffolds assembled by HFFs cultured in L-ascorbic acid-free and serum-free culture medium.**

(a) Experimental timeline: one day of cell attachment followed by four days of culture during which human foreskin fibroblasts (HFFs) were exposed to standard L-ascorbic acid-free and serum-free culture medium (native HFFs) or TGFβ1-enriched, L-ascorbic acid-free and serum-free culture medium (TGFβ1-treated HFFs). Alexa 488-labeled Fn was added to the culture medium in both groups. (b) Native and TGFβ1-treated HFFs both assembled an Fn-rich ECM on account of the supplied plasma Fn in the culture medium. Type-1 collagen fibrils were not observed in the ECM via immunostaining in either group, due to the absence of L-ascorbic acid and serum in the culture medium. Scale bars: 50 μm. (c) ECM quantification was performed as described for Figure 1c. TGFβ1 supplementation significantly increased Fn assembly (*: p < 0.0001), but did not increase collagen assembly. Statistical comparisons via one-way ANOVA with Sidak’s multiple comparisons test, significance set at p < 0.05 for all comparisons.


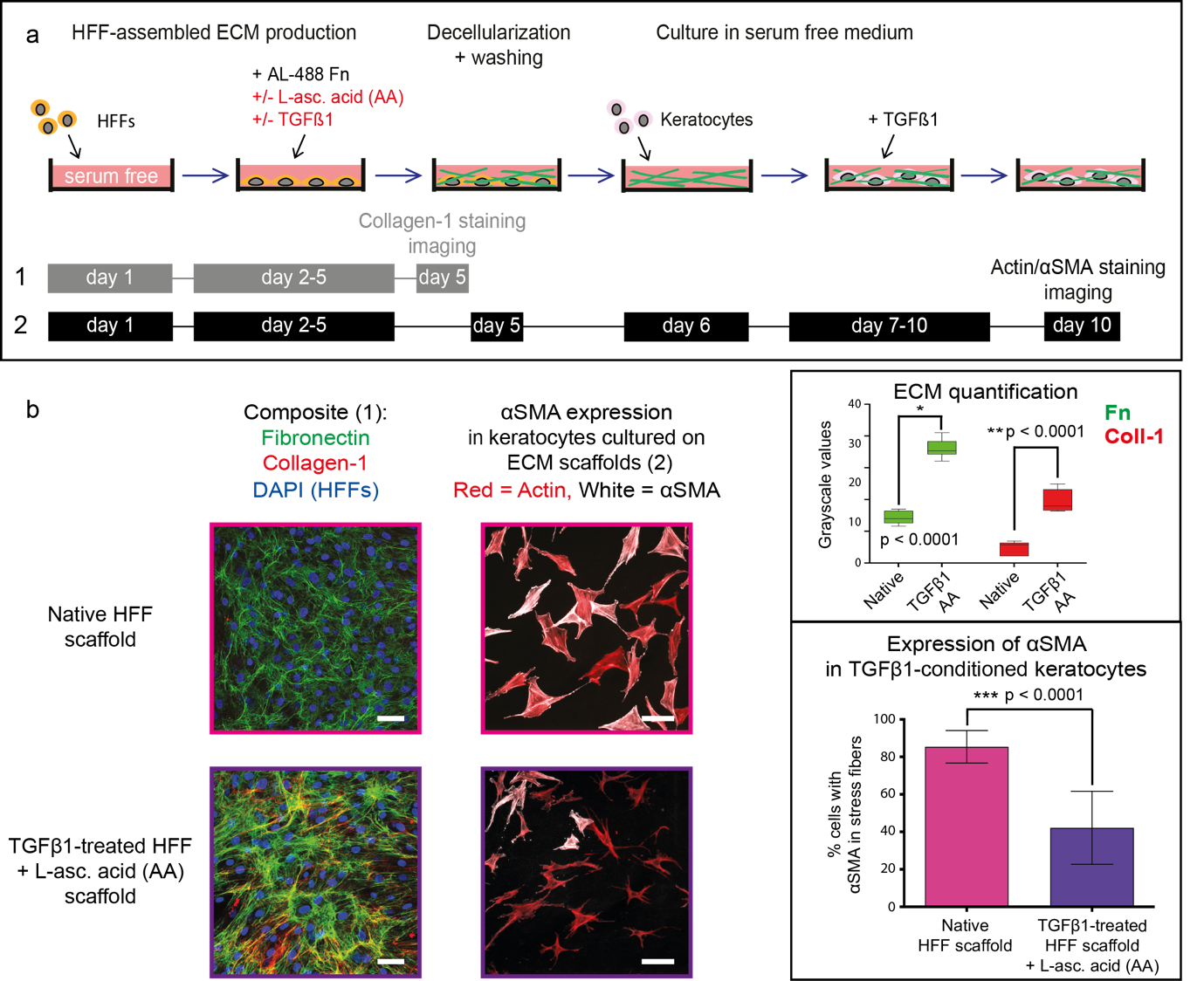


**Figure S14: αSMA expression by TGFβ1 conditioned keratocytes is decreased on myofibroblast-derived ECM scaffolds with fibrillar collagen-1.**

(a) Experimental timeline: human foreskin fibroblasts were cultured in serum-free culture medium without L-ascorbic acid added (native HFFs) or in identical culture medium supplemented with L-ascorbic acid and TGFβ1 (TGFβ1-treated HFFs + L-asc.acid) to allow a four day period of ECM assembly. A subset of ECM scaffolds was imaged after Collagen-1 immunostaining. The rest of the scaffolds underwent decellularization and further processing as detailed in Fig 5a. (b) Left column and box plot: native (violet) and L-ascorbic acid + TGFβ1 supplemented (purple) HFFs both assembled a Fn-rich ECM on account of the supplied plasma Fn in the culture medium. ECM quantification was performed as described for Figure 1c. Combined supplementation of TGFβ1 and L-ascorbic acid significantly increased both Fn (*: p < 0.0001) and collagen (**: p < 0.0001) assembly. Scale bars: 50 μm. Middle column and bar chart: αSMA incorporation into stress fibers was observed in 85% of TGFβ1 conditioned keratocytes on native HFF-assembled ECM scaffolds (violet), compared to 42% on L-ascorbic acid + TGFβ1 supplemented HFF- assembled ECM scaffolds (purple) (***: p < 0.0001). Scale bars: 100μm. Measurements from >250 cells per scaffold type were included in the bar chart: bars signify the means and whiskers the standard deviation from the mean. Statistical comparisons were performed via one-way ANOVA with Sidak’s multiple comparisons test (Fn/Collagen-1) and unpaired t-tests (αSMA), with significance set at p < 0.05 for all comparisons.


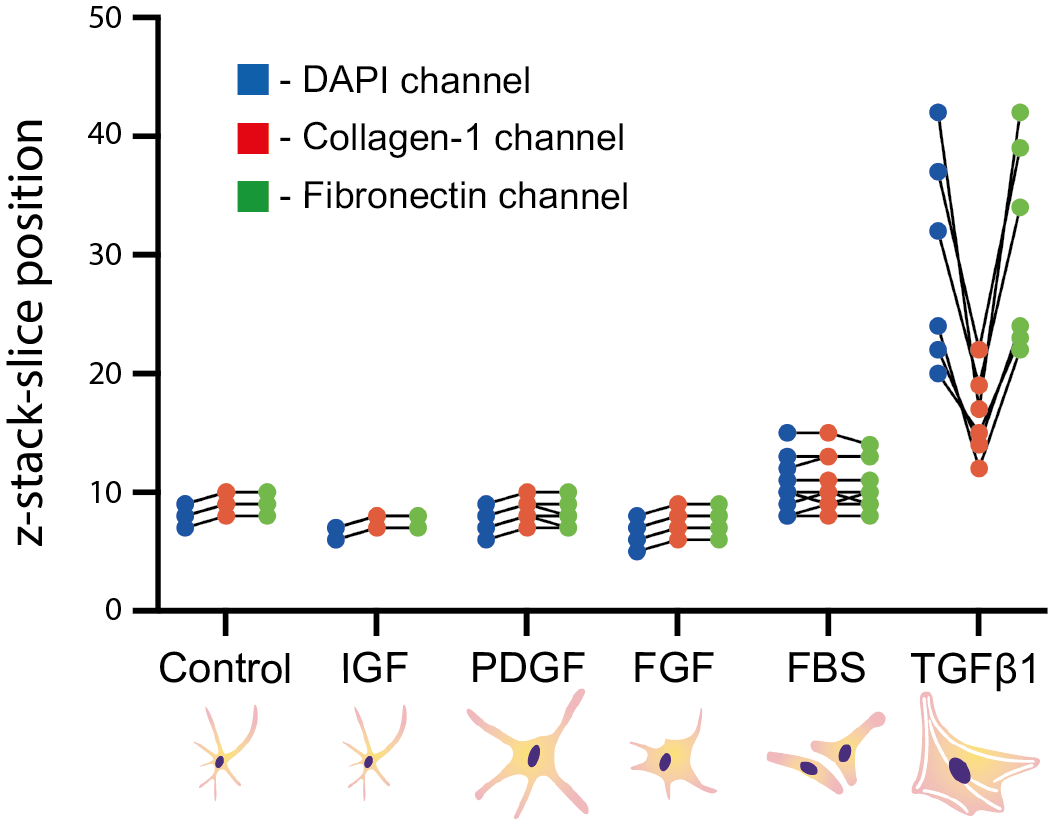
**Figure S15: Maximum intensity z-stack slice position Fibronectin and Collagen-1 ECM in growth factor conditioned keratocytes.**

As z-dimensions varied within and between groups, a substack consisting of three continuous z-stack-slices was defined around the z-stack-slice with maximum fluorescence intensity in the collagen-1 and Fn-channels. The z-position of these maximum intensity z-stack-slices is plotted in this graph, and varied significantly between the collagen-1 and Fn channel in the TGFβ1-treated samples only.

Methods

***Primary rabbit keratocyte isolation and cell culture***

Eyes used for isolation of primary corneal keratocytes were obtained from healthy rabbits at a local abattoir. The anterior segment of all eyes was found to be normal after examination with a focal light source. The eyes were immediately placed in Dulbecco’s minimum essential medium (DMEM) GlutaMAXX + Pyruvate (Cat# 21885-025, Life Technologies, Zug, Switzerland), kept on ice and processed within 6 hours after tissue harvesting. The keratocytes were isolated using previously described methods^4^.

Keratocytes were cultured at 37°C in 5% CO_2_ on collagen coated, plasma treated polystyrene culture dishes (tissue culture plastic) in serum free medium (DMEM supplemented with 1% RPMI 1640 Vitamins Solution, Cat# R7256, Sigma; 1% NEAA, Cat# 11140-050, Life technologies; 0.11 mg/ml L-ascorbic acid, Cat# A-8960, Sigma; 1% Penicillin & Streptomycin, Cat# 15140, Invitrogen). The supplementation of ascorbate is necessary for the production and stabilization of procollagen and the collagenous ECM in these cultures^5^. Keratocytes were exposed to specific growth factors (IGF-I: 10 ng/ml, Cat# 100-11; PDGF-BB: 50 ng/ml, Cat# 100-14B; FGF-2: 10 ng/ml, Cat# 100-18B; TGFβ1: 5 ng/ml, Cat# 100-21C, Peprotech, Hamburg, Germany) or 10% Fetal bovine serum (FBS, Cat# 12662-029, Invitrogen) supplemented to the medium for a minimum of four days to induce the various growth factor-stimulated cell phenotypes^4^. Exposure to FBS was performed as control, since FBS is a common cell culture medium supplement rich in an undefined variety and quantity of growth factors, hormones, proteins and nutrients. Trypsin used for passaging keratocytes was neutralized with Soybean Trypsin Inhibitor (Cat# 17075-029, Life technologies) at a concentration of 0.5 mg/ml in 1x phosphate buffered saline (PBS). The growth factor concentrations represent the lowest concentrations with a maximal effect on cell morphology and F-actin organization. These concentrations were adopted from previous studies^4, 6^. TGFβ1 was supplemented at 5 ng/ml. For interventional experiments, cells were seeded onto glass cell culture substrates with adsorbed unlabeled Fn. The cell culture surfaces were incubated with 25 ug/ml unlabeled Fn for 1 h at room temperature (or overnight at 4°C), followed by a 15 min. UV sterilization.

***Primary human foreskin fibroblast (HFF) cell culture***

Primary human foreskin fibroblasts (HFF; C-12300, Promocell, Germany) were cultured using previously described protocols^7^. Briefly, the HFFs were maintained at 37 °C with 5% CO2 in fibroblast growth medium (C-23010, Promocell, Germany). This medium does not contain serum but does contain 1 ng/ml recombinant human fibroblast growth factor and 5 μg/ml insulin for more consistent cell culture results. After reaching 70–90% confluency, HFFs were passaged at least once before being deployed in experiments. HFFs below passage 10 were used in all experiments to prevent the spontaneous appearance of myofibroblasts, which was observed in cultures at higher passage numbers.

***Immunocytochemistry***

Primary antibodies:

Mouse monoclonal anti-α-SMA (O.N.5, Cat# ab 18147 [1A4], Abcam, Cambridge, UK); 1:100 dilution in 1% BSA/PBS for 1 hour, used as myofibroblast phenotype specific marker.

Mouse monoclonal anti-transglutaminase 2 antibody (CUB 7402], Abcam); 1:100 dilution in 1% BSA/PBS for 1 hour.

Goat polyclonal anti-Fibronectin, N‐20 (Cat# sc‐6953, Santa Cruz, CA; 1/100 dilution in 3%BSA/PBS prior to cell permeabilization for 1 hour)

Mouse Monoclonal anti-Collagen I ([COL-1], Cat# ab 90395, Abcam, Cambridge, UK; 1/200 dilution in culture medium for 1 hour at 37C)

Secondary antibodies:

Goat anti-mouse-Alexa Fluor 555 (Cat# A21424, Life technologies; 1:100 dilution in 1% BSA/PBS for 30 minutes).

Donkey anti-mouse Alexa Fluor 488 (Cat# A-21202, Invitrogen; 1:100-1000 dilution in 3% BSA/PBS for 1 hour).

Donkey anti-goat-Alexa Fluor 633 (Cat# A21082, Life Technologies; 1:100 dilution in 3% BSA/PBS for 1 hour).

Compartment specific fluorescent dyes:

Phalloidin-Alexa Fluor 488 and 568 (Cat # A12379, Cat # A12380, Invitrogen/Life technologies; 1:100-200 dilution in 1x PBS +/- 1-3% BSA for 2 hours) were used as cellular F-actin and actin stress fiber markers.

4',6-diamidino-2-phenylindole (DAPI) (Cat # D3571, Invitrogen/Life technologies; 1:1000 dilution in 1x PBS for 10-15 minutes) was used to counterstain cell nuclei.

Routine staining protocol:

Cells were fixed in 3.7% formaldehyde (Formaldehyde, Cat# 18814, Polysciences Inc., Warrington, PA, USA) in 1x PBS for 5-15 minutes and incubated in 0.5% BSA (Cat# A9418, Sigma) and 0.1% Triton-X-100 (Cat# X100, Sigma) for 10 minutes to block non-specific protein binding and to permeabilize the cells. All antibodies and compartment specific fluorescent dyes were added after fixation and permeabilization, with the exception of the primary anti-Coll-1 antibody and the primary anti-Fn antibody, which were added to the live cell culture at 37C and after fixation but prior to permeabilization, respectively. The cells were washed three times with 1x PBS between each step and all steps were performed at room temperature. Finally, samples were left in 1x PBS until immunofluorescent imaging with a Zeiss Axiovert 200M epifluorescent microscope (Carl Zeiss Imaging Solutions GmbH, Munich, Germany) or confocal microscope (Olympus FV-1000, Leica SP5).

For single cell imaging experiments the cells were seeded at a density of 5000 cells/cm^2^. Due to their high proliferation rates FBS induced keratocytes were seeded at an initial cell density of 1000 cells/cm^2^.

***Image analysis for ECM quantification***

To ensure robust results, quantification of immunofluorescence intensities in Z-stack confocal microscopy data was carried out using a custom-built FIJI macro, which can be accessed on GitHub (<https://github.com/BennSynergy/FIJI-macro_zStackQuant.git>, DOI: 10.5281/zenodo.7978897)^8^. For each dataset, the mean fluorescence intensity of each z-stack-slice was plotted over the entire z-distance to visualize the fluorescence signal distribution. It was noted that the z-position of z-stack-slices exhibiting maximum fluorescence intensity significantly varied between the collagen-1 and Fn channel in TGFβ1-treated samples (see Supplementary fig. S15). Consequently, quantification of Fn and collagen-1 fluorescence intensities was performed in a 3-slice substack that surrounded the z-stack-slice with peak fluorescence intensity in the collagen-1 channel. This strategy was adopted since the selection of the maximum z-position from either the collagen-1 or Fn channel for fluorescence intensity quantification did not alter the relative differences observed among the interventional groups. For each dataset, the mean intensity of the 3-slice substack was calculated and used for further statistical analyses. Plots were constructed from data extracted from a total of 6-10 samples per phenotype divided over two separate experiments. The mean DAPI, Fn and Collagen-1 fluorescence intensity of each z-stack-slice was plotted over the entire z-distance to visualize the fluorescence signal distribution of a representative sample of each cell phenotype (Fig. 1c).

***MTT assay***

The assay was performed according to the manufacturer’s instructions. Briefly, cell metabolic activity was measured via MTT assay (Cell Proliferation Kit I (MTT), Cat # 11465007001, Roche) as an indicator of cell proliferation in growth factor conditioned keratocytes at culture day 5 and in growth factor deprived keratocytes at culture day 9. The cells were trypsinized and seeded in triplicates in 96-well plates in 100 μl of their respective growth factor supplemented media. After 24h, the MTT reagent (1:10) was added to the cells and incubated for 4h at 37°C, 5% CO_2_. During this time, metabolically active cells cleave the tetrazolium salts contained in the MTT reagent into formazan, which has a higher absorbance than the tetrazolium salts. Then, 100 μl of the solubilization solution was added to each well and the plate incubated overnight at 37°C, 5% CO_2_. Finally, the absorbance of the medium was measured with a plate reader (Tecan M200). Absorbance values were normalized to that of the control keratocytes in serum-free medium and reported in Fig. S4.

***Real-time PCR evaluation of keratocyte and myofibroblast markers***

Cells were lysed and RNA was isolated using manufacturers protocol (Nucleospin RNA-II, Cat # 740955.50, Macherey Nagel AG, Oensingen, Switzerland). A spectrophotometer was used to determine RNA yield (Nanodrop; Thermo Scientific, Wilmington). cDNA was produced using manufacturers protocol (Taqman® Reverse transcription Reagent, Cat# N808-0234, Applied Biosystems). Gene expression was evaluated by real-time PCR as previously published^1^. Briefly, real-time PCR was performed using SYBR green reagents (Sensimix SYBR kit from Bioline, Cat# QT605-05, Biocompare, San Francisco, CA, USA) and validated real-time PCR primers for rabbit keratocyte and myofibroblast markers (Table 1, supporting information). An annealing temperature of 63°C was used. Products were evaluated by melt curve analysis. Relative quantification was performed by the ΔΔCT method with β-actin used as normalizing housekeeping gene. The experiment was performed in triplicate with duplicate samples, with the results of one representative experiment shown in supplementary figure S3. The data from all three experiments were pooled for statistical comparison.

***Nanopillar array fabrication***

Nanopillar platforms were fabricated exploiting nanosphere lithography followed by a molding process using previously described protocols^9^. The photoresist SU-8 nanopillars measured 0.25 μm in diameter and 1.5 μm in height with a 0.8 μm pillar center to pillar center distance. The spring constant of a representative SU-8 nanopillar was measured by Atomic Force Microscopy (AFM) by deflecting single nanopillars with the AFM cantilever. A spring constant of 78.8 ± 3 nN/µm for single nanopillars was generated from the slope of the AFM force-displacement curves and was used to calculate the cell-generated horizontal traction forces on the pillar substrate. The pillar substrates were first passivated with Pluronic F-127 (P2443, Sigma) solution to prevent cell adhesion to the sides of the nanopillars. Plasma Fn (25 μg/ml, 1h at RT) was then applied to the pillar tops by contact printing to allow cell adhesion to the top of the pillars only. These Fn-coated nanopillars were biocompatible and allowed a natural spreading of cells on top of the nanopillars.

***Cell traction force measurement***

After trypsinization cells were seeded onto nanopillar substrates with a density of 2500 cells/cm^2^. Cells were fixed 3 hours after seeding, then phalloidin stained and imaged with a 63X oil immersion objective (NA=1.43) on a confocal microscope (Leica SP5). This timeframe was chosen since we aimed to measure force generation after the initial cell to substrate attachment phase^10^, but prior to any significant ECM deposition. The pillar displacement underneath the cells in xy direction was quantified by comparing two sets of images with focal planes at the pillar base and top, respectively. DIC (nanopillar) and fluorescent (cell edge) images were recorded and the pillar displacement was analyzed with particle tracking software (Diatrack 3.03, Powerful Particle Tracking, Semasopht; and Fiji, plugin, template matching for drift collection). The traction forces by which the cells displaced the nanopillars were calculated according to Hooke’s law: F = k*x, given the pillar spring constant of k = 78.8 ± 3 nN/µm. Since force generation measurements on fixed cells reflect the cellular contractile state at a single point in time and cellular force generation changes over time as adhesion complexes mature ^10^ we were interested in force generation measurements over time. To visualize cell edges in live-cell imaging experiments the cells were incubated with a fluorescent membrane dye (Vybrant/ Dil, Invitrogen, 1:200 dilution in culture medium) for 10 minutes in suspension prior to seeding. Within three hours after seeding, each cell was imaged for 30 min with a scanning ratio of 1 frame/min while being kept at 37 ºC and 5% CO_2_ on an incubated microscope stage.

***Scanning electron microscopy***

Cells on nanopillar arrays were imaged using a Scanning Electron Microscope (SEM, Zeiss ULTRA 55) after fixation and critical point drying using standard protocols^9^. Briefly, this stepwise dehydration procedure started by adding 0.5% aqueous solution of polyethylenimine, followed by dehydrating the samples with a series of ethanol-water washes (25%, 50%, 75%, two 95%, and 100% ethanol) and finishing by drying the sample using critical point drying equipment and CO_2_. After the dehydration process, the samples were sputter-coated with a 5 nm thick layer of gold.

***Direct stochastic optical reconstruction microscopy (dSTORM)***

For dSTORM imaging, keratocytes (5.500 cells/cm^2^), preconditioned with 5 ng/ml TGFβ1 for 4 days, were seeded onto coverslips coated with 50 µg/ml arginine-eluted Fn in PBS for 1h at RT. After a 3h incubation in TGFβ1 supplemented medium and 3 washes in PBS, the samples were fixed in 4% PFA in PBS for 10 minutes and again washed three times with PBS. Samples were permeabilized with 0.1% (v/v) Triton X-100 and 0.5% (w/v) BSA in PBS for 10 minutes and then blocked for an additional 10 minutes with 3% (w/v) BSA in PBS at RT. Actin cytoskeleton staining was performed by incubating with Alexa Fluor 647 phalloidin at 1:20 dilution for 30 minutes. After 3 washes in PBS, samples were imaged using a home-built set-up for single-molecule localization microscopy, as previously described^11^.

***Preparation of cell derived ECM scaffolds***

Tissue equivalents, often collagen gels seeded with fibroblasts, were used to investigate ECM stress regulatory principles in most previous studies^6, 12, 13^. Although collagen fibrils can self-assemble *in vitro*, their assembly and proper organization *in vivo* are regulated by many additional binding partners, including cellular fibronectin and integrins^14^. Furthermore, resident tissue fibroblasts control the supramolecular fibril organization within and the three-dimensional structure of the collagen matrix^15^. Cultured human foreskin fibroblast (HFF) assembled ECM scaffolds were therefore used here to provide a more physiologically relevant 3D cell culture environment to evaluate the influence of the ECM on K-F/M transition.

HFFs (50,000 cells/cm^2^) were seeded onto Fn coated surfaces and allowed to adhere for 30 minutes. The culture medium was then replaced by cell-type specific medium containing FRET labeled Fn or Alexa 488 singly labeled Fn (45ug/ml unlabeled Fn and 5ug/ml labeled Fn). Cells were cultured for 4 days with a medium change after 48 hours prior to imaging.

***Fn isolation and labeling***

Fn was isolated from human plasma (Zurcher Blutspendedienst SRK, Switzerland) by affinity chromatography as previously described^2^. Double labeling of plasma Fn with Alexa Fluor® 488 carboxylic acid, succinimidyl ester (Cat A-20000, Molecular Probes/Invitrogen) as donor on amines and Alexa Fluor® 546 carboxylic acid, succinimidyl ester (Cat A-20002, Molecular Probes/Invitrogen) as acceptor on free sulfhydryls was performed as previously described^2^.

***Image acquisition and analysis for Fluorescent Resonance Energy Transfer (FRET)***

Fluorescent Resonance Energy Transfer (FRET) analysis was performed as previously described^2^. All FRET images were acquired from living cell samples, except for the FRET images from HFF derived ECM scaffolds for keratocyte reseeding experiments and for decellularization experiments, which were acquired from decellularized samples. FRET I_A_/I_D_ ratios were calibrated to different Fn conformations in PBS and various strength GdnHCl solutions. Dimeric and fully folded Fn in PBS showed strong energy transfer whereas monomeric and significantly unfolded Fn-FRET in 4M GdnHCl showed dramatically decreased energy transfer. According to previous studies on Fn conformations in solution ^2^, the I_A_/I_D_ value of monomeric Fn-FRET in 1M GdnHCl will be used to indicate the very first onset of loss of secondary structure. To measure the FRET ratio, all images were acquired using an Olympus FV-1000 scanning laser confocal microscope with a 1.35NA 60X oil immersion objective. Alexa Fluor 488 donors were excited with a 488 nm laser. Emitted light was split using a 50/50 beam splitter and the signals from the donor and acceptor channels were detected in two separate photomultiplier tubes with emission detection window settings of 514–526 nm (donor channel) and 566–578 nm (acceptor channel). Images from five regions of interest (ROIs) were acquired from each sample at a resolution of 512 × 512 pixels. In each region, FRET images were acquired throughout the entire Z-stack with a 1 μm slice-to-slice distance. Images were then analyzed using Matlab (http://www.mathworks.com/) with a self-programmed script (script available as supplementary information). Decreasing Fn-FRET I_A_/I_D_ ratios indicated more extended Fn conformations. Histograms were plotted from all data pixels and Fn-FRET I_A_/I_D_ ratios were color-coded within the range of 0.4 to 1.0 to produce FRET images^2^.

***Seeding native keratocytes onto HFF-derived ECM scaffolds.***

Cell-derived ECM scaffolds assembled by fibroblasts (native HFFs) and myofibroblasts (TGFβ1-treated HFFs) were isolated. After 4 days, the cell monolayer was decellularized by a previously described protocol to yield a cell-free cell derived ECM scaffold^16^. Briefly, samples were rinsed twice with warm PBS (pH 7.4), incubated in 300 μl warm extraction buffer (20 mM NH_4_OH solution in PBS (pH = 9.95) with 0.5% (v/v) Triton X-100) for for 3 min.. Samples were then rinsed three times with PBS (pH 7.4). Keratocytes were then seeded onto the acellular matrix at a density of 10,000 cells/cm^2^. After cell attachment, 5ng/ml TGFβ1 was added to the serum-free keratocyte culture medium to induce myofibroblast differentiation of the keratocytes. Cells were cultured for 4 days with a medium change after 48 hours prior to imaging. The cells were then stained with phalloidin and αSMA and imaged with an inverted epifluorescence microscope (Axiovert 200M; Carl Zeiss MicroImaging, Inc.). During image analysis, the thresholding tool in ImageJ (version 2.0.0-rc-69/1.52p), set on ‘Default’, was used to produce binary images used for counting the number of cells with αSMA positive stress fibers and the number of actin stress fiber expressing cells. The lower threshold was set at 2200 and at 2500 in the αSMA channel and in the actin channel, respectively. The upper threshold was set at the maximum in both channels. The proportion of cells expressing αSMA in their stress fibers was calculated and compared between different groups. The bar charts were constructed from cell counts (>200 cells per scaffold type) averaged over ten random fields of view from one representative experiment in a series of three per group.

***Statistics:***

The presence of significant differences between any groups was evaluated by unpaired t-tests or one-way ANOVA for parametric data and by Kruskal–Wallis one-way ANOVA for nonparametric data. Post-hoc tests were performed where applicable to identify significant differences between specific groups: Holm-Sidak’s multiple comparison test for αSMA incorporation into stress fibers, Dunn’s multiple comparison test for the MTT, rt-PCR, and keratocyte Fn ECM quantification assays, Tukey’s or Sidak’s multiple comparison test for the ECM Fn strain (FRET), Tukey’s multiple comparison test for the keratocyte Collagen-1 ECM quantification and the single cell force generation experiments, and Sidak’s multiple comparison test for the HFF Fn and Collagen-1 ECM quantification assays. The level for statistical significance was set at P < 0.05 for all comparisons. GraphPad Prism version 6.00 for Windows (GraphPad Software, La Jolla CA, USA, www.graphpad.com) was used for all statistical analyses. The αSMA, rt-PCR and FRET experiments were performed in triplicate and the results of one representative experiment were presented. Per phenotype, αSMA incorporation into stress fibers was evaluated in over 180 cells in ten random fields of view from one representative experiment. Cells were pooled from three experiments and 18 cells per phenotype were evaluated in the cellular contractility experiments. An average displacement value per nanopillar was calculated per cell as overall measure for cellular contractility.

Custom-built FIJI script for ECM quantification

The custom FIJI macro developed for the image analysis in this study is available on GitHub at <https://github.com/BennSynergy/FIJI-macro_zStackQuant.git> (DOI: 10.5281/zenodo.7978897)^8^.

MATLAB script for FRET analysis

Please see separate supplementary txt. file document ‘FRETNoZStack’.

References

1. Jester, J.V., Brown, D., Pappa, A. & Vasiliou, V. Myofibroblast differentiation modulates keratocyte crystallin protein expression, concentration, and cellular light scattering. *Invest Ophthalmol Vis Sci* **53**, 770-778 (2012).

2. Smith, M.L. *et al.* Force-induced unfolding of fibronectin in the extracellular matrix of living cells. *PLoS biology* **5**, e268 (2007).

3. Ritter, S.J. & Davies, P.J. Identification of a transforming growth factor-beta1/bone morphogenetic protein 4 (TGF-beta1/BMP4) response element within the mouse tissue transglutaminase gene promoter. *The Journal of biological chemistry* **273**, 12798-12806 (1998).

4. Jester, J.V., Barry-Lane, P.A., Cavanagh, H.D. & Petroll, W.M. Induction of alpha-smooth muscle actin expression and myofibroblast transformation in cultured corneal keratocytes. *Cornea* **15**, 505-516 (1996).

5. Chan, D., Lamande, S.R., Cole, W.G. & Bateman, J.F. Regulation of procollagen synthesis and processing during ascorbate-induced extracellular matrix accumulation in vitro. *The Biochemical journal* **269**, 175-181 (1990).

6. Kim, A., Lakshman, N., Karamichos, D. & Petroll, W.M. Growth factor regulation of corneal keratocyte differentiation and migration in compressed collagen matrices. *Invest Ophthalmol Vis Sci* **51**, 864-875 (2010).

7. Zhang, Y. *et al.* Disentangling the multifactorial contributions of fibronectin, collagen and cyclic strain on MMP expression and extracellular matrix remodeling by fibroblasts. *Matrix biology : journal of the International Society for Matrix Biology* **40**, 62-72 (2014).

8. Benn, MC. FIJI-macro_zStackQuant. <https://github.com/BennSynergy/FIJI-macro_zStackQuant.git>, DOI: 10.5281/zenodo.7978897.

9. Shiu, J.Y., Aires, L., Lin, Z. & Vogel, V. Nanopillar force measurements reveal actin-cap-mediated YAP mechanotransduction. *Nature cell biology* **20**, 262-271 (2018).

10. Dubin-Thaler, B.J. *et al.* Quantification of cell edge velocities and traction forces reveals distinct motility modules during cell spreading. *PloS one* **3**, e3735 (2008).

11. Fruh, S.M., Schoen, I., Ries, J. & Vogel, V. Molecular architecture of native fibronectin fibrils. *Nature communications* **6**, 7275 (2015).

12. Petroll, W.M., Varner, V.D. & Schmidtke, D.W. Keratocyte mechanobiology. *Exp Eye Res* **200**, 108228 (2020).

13. Jester, J.V. & Ho-Chang, J. Modulation of cultured corneal keratocyte phenotype by growth factors/cytokines control in vitro contractility and extracellular matrix contraction. *Exp Eye Res* **77**, 581-592 (2003).

14. Kadler, K.E., Hill, A. & Canty-Laird, E.G. Collagen fibrillogenesis: fibronectin, integrins, and minor collagens as organizers and nucleators. *Current opinion in cell biology* **20**, 495-501 (2008).

15. Kadler, K.E. Fell Muir Lecture: Collagen fibril formation in vitro and in vivo. *Int J Exp Pathol* **98**, 4-16 (2017).

16. Antia, M., Baneyx, G., Kubow, K.E. & Vogel, V. Fibronectin in aging extracellular matrix fibrils is progressively unfolded by cells and elicits an enhanced rigidity response. *Faraday discussions* **139**, 229-249; discussion 309-225, 419-220 (2008).
