# Supplementary material for: Growth factors and mechano-regulated reciprocal crosstalk with extracellular matrix tune the keratocyte–fibroblast/myofibroblast transition
Source: Sci Rep. 2023 Jul 13;13:11350. doi: 10.1038/s41598-023-37776-9 (PMC10345140; doi:10.1038/s41598-023-37776-9)
Supplement: Supplementary file 2 — Supplementary Information 2. [file 41598_2023_37776_MOESM2_ESM.pdf]

```
function Newtest

warning off;

f1=1;
f2=1;

extension = '.tif'; % file extension
overlay_histogram = strcat('output\histogram.eps');

mkdir ('output\'); % make output folder
dc1 = xlsread('D:\WorkPlace\bg.xls', 'Sheet1', 'A1'); % get
background intensity
dc2 = xlsread('D:\WorkPlace\bg.xls', 'Sheet1', 'B1');
counter = 1;

file_exit=2;

    xlswrite('output\FN-FRET-no-Z-stack.xls', {'Average
Mean','Mode','Standard
Deviation','Avgirsize','Perc','','','','','Per5','Per95','Z-
Position'}, 'Result', 'B1'); % label the first row

while file_exit>0


    filename = strcat(int2str(f1),'-',int2str(f2)); % get filename
    folder = strcat('D:\WorkPlace\',filename,'.oif.files');

    % generate path of the input / output files
    path1 = strcat(folder,'\s_C001',extension);
    path2 = strcat(folder,'\s_C002',extension);
    savefile = strcat('output\',filename,'.eps');

    % check if image exist
    file_exit = exist(path1);

    % if exist, process it
    if file_exit ~= 0

        counter = counter+1;

        %BRING IT ON!
        warning('off', 'all');  %DISABLE THIS LINE IF YOU NEED TO
DEBUG!!!!!!!
        ac1 = imread(path1);
        do1 = imread(path2);
        accThresh = 50; donThresh = 50;
        upLimit = 0.8; lowLimit = 0.3; limits = [lowLimit upLimit];

        %number of bins in ir histogram
        z = 90; binsize = (upLimit-lowLimit)/z;
        %percentage data below ??M input
        M0 = .95; M1 = .62; M1DTT = .55; M2 = .53; M4 = .44;
```

```
        %convert to double precision
        ac1 = double(ac1); do1 = double(do1);

        %averaging mask
        ac1 = colfilt(ac1,[3 3],'sliding',@mean); do1 = colfilt(do1,
[3 3],'sliding',@mean);

        %Remove dark current offset
        ac1 = ac1-dc1; do1 = do1-dc2;

        %compensate for 50_50BS intensity attenuation
        do1 = do1*1.09;

        %eliminate low intensity or saturated pixels
        threshIndAcc = find(ac1 <= accThresh | ac1 >= 3700);
threshIndDon = find(do1 <= donThresh | do1 >= 3700);
        ac1(threshIndAcc) = 0; do1(threshIndDon) = 0;

        %fret calculation
        ir = ac1./do1;

        %Remove un-realistic ir values and NaNs
        realInd = find((isnan(ir) | ir < lowLimit | ir > upLimit));
        ir(realInd) = 0;

        %determine number of ir pixels and ir statistics
        avgirArray=nonzeros(ir);
        avgirsize=size(avgirArray);
        avgirmean=mean(avgirArray);
        avgirstd=std(avgirArray);
        per5 = prctile(avgirArray,5); per95 = prctile(avgirArray,
95);
        r0 = find(avgirArray < M0); r1 = find(avgirArray < M1);
r1dtt = find(avgirArray < M1DTT); r2 = find(avgirArray < M2); r4 =
find(avgirArray < M4);
        perc(1)=length(r4)/avgirsize(1,1)*100; perc(2)=length(r2)/
avgirsize(1,1)*100; perc(3)=length(r1dtt)/avgirsize(1,1)*100;
perc(4)=length(r1)/avgirsize(1,1)*100; perc(5)=length(r0)/
avgirsize(1,1)*100;

        %generate a histogram for ir mode determination and display
        bins = lowLimit:binsize:upLimit;
        [histCounts, histX] = hist(avgirArray,bins);
        [C,I] = max(histCounts);
        modeReal = histX(I);  %Calculate mode from histogram data
        histCounts = histCounts/C;  %normalize the image to scale
from 0.0-1.0

        %Plotting
        figure(1);
        subplot(3,1,[1 2]); imagesc(ir, limits);
        axis off; axis square; colorbar('horiz'); set(gca,
'FontName', 'Times New Roman', 'FontSize', 12);
```

```
        subplot(3, 1, [3], 'replace'); line(histX, histCounts);
        xlabel('intensity ratio (acceptor/donor)', 'FontName',
'Times New Roman', 'FontSize', 12);
        ylabel('events', 'FontName', 'Times New Roman', 'FontSize',
12);
        set(gca, 'FontName', 'Times New Roman', 'FontSize', 12);
axis([lowLimit upLimit 0 1]);

        figure(2);
        line(histX, histCounts);
        xlabel('intensity ratio (acceptor/donor)', 'FontName',
'Times New Roman', 'FontSize', 12);
        ylabel('events', 'FontName', 'Times New Roman', 'FontSize',
12);
        set(gca, 'FontName', 'Times New Roman', 'FontSize', 12);
axis([lowLimit upLimit 0 1]);

        %save the final data as a .tiff
        saveas(figure(1), savefile, 'psc2');
        saveas(figure(2), overlay_histogram, 'psc2');

        %output data for further analysis into a text file

        output = [ avgirmean modeReal avgirstd avgirsize(1,1) perc
per5 per95];

        % export results to excel files
        row = strcat('B',int2str(counter));
        xlswrite('output\FN-FRET-no-Z-stack.xls', output, 'Result',
row);


        f2=f2+1;
        disp(savefile);
    else
        if f2==1
            disp(counter);
        else
            file_exit=1;
            f1=f1+1;
            f2=1;
        end
    end
end
end
```
